# Nation-based peer assessment of Europe’s Sustainable Development Goal performance

**DOI:** 10.1371/journal.pone.0287771

**Published:** 2023-06-28

**Authors:** Enda Murphy, Patrick Paul Walsh, Ethan Murphy

**Affiliations:** 1 School of Architecture Planning and Environmental Policy, University College Dublin, Dublin, Ireland; 2 Vice-President for Education, UN Stainable Development Solutions Network, New York, NY, United States of America; 3 School of Politics and International Relations, University College Dublin, Dublin, Ireland; 4 School of Behavioural and Health Sciences, Australian Catholic University, Melbourne, Victoria, Australia; Universiy of Belgrade, Faculty of Transport and Traffic Engineering, SERBIA

## Abstract

Less than seven years remain for Europe to meet the targets of the United Nations Sustainable Development Goals (SDGs). However robust and accurate methods for assessing SDG progress are currently lacking. Through the development of several SDG indices, this study addresses this critical knowledge gap by providing the means to accurately identify national ’problem areas’ and thereby accelerate SDG achievement. Specifically, an indicator-based approach has been used to create a composite index containing 166 unique SDG indicators that assess a nation’s SDG performance compared to the best and worst performers in the European Union (EU). Our results indicate that each EU nation is on average, 58% of the way towards the best performer in the overall SDG indicator framework. A nuanced taxonomy has been developed that allows for the assessment of SDG performance in several critical dimensions of the SDGs, including in ’Means-of-Implementation (MoI)’, ’Linkage’, and ’Outcome’ indicators. The index’s comprehensive framework allows for EU’s performance in individual SDG indicators to be investigated while providing the most accurate assessment of national SDG performance, to date. Overall, the indices presented in this paper can significantly enhance the understanding of SDG performance while concurrently guiding national and EU SDG policy development.

## 1 Introduction

Adopted in 2015 by 193 member states, the United Nations’ Sustainable Development Goals (SDGs) represent the most prominent example of an international collaborative initiative to achieve a set of time-sensitive sustainable development targets [1]. With only seven years remaining until the 2030 achievement target and the world failing to progress towards the SDGs for the second year in a row [2], the probability of attaining the SDGs is diminishing. Indeed, the stability of the Earth as a system is currently at risk as five of the nine planetary boundaries–describing the environmental limits within which humanity as a species may continue to thrive [3]—have now been exceeded [4]. Furthermore, Earth’s biodiversity crises are proceeding at alarming rates [5]. A recent report from the Worldwide Fund for Nature (WWF) has found that Earth’s species have experienced an average reduction in numbers of 69% between the years 1970 and 2018 [6]. Humanitarian crises are also mounting as the number of individuals who have been forcibly displaced from their homes now exceeds 100 million, reaching levels similar to that seen during the Second World War [7]. In addition, the SARS-CoV-2 pandemic has reversed years of progress in reducing global poverty and food insecurity [8–10]. Various scientists have recognised that global transformative changes are now necessary for the world to be redirected towards sustainable development [11, 12].

Effective evidence-based policy-making is essential to SDG achievement [13]. As such, progress should be guided by robust statistical metrics that accurately describe a country’s advancement towards SDG achievement while also allowing for the identification of best practices [2]. However, the scale and complexity of the SDG indicator framework render it difficult to holistically monitor progress towards the 169 SDG targets using one or a small number of its 231 unique indicators [14]. As a consequence, several organisations have developed composite indices that reduce the complexity of SDG reporting and allow for the assessment of country-specific SDG performance [2, 15, 16]. Through benchmarking a nation’s performance, composite indices aim to facilitate policy-tracking, raise public awareness, and influence future policy-making [17]. However, previous composite indices have been critiqued for lacking practical value [18–20]. Within this context, this paper presents an index methodology that attempts to bridge the gap between analytics and assessment metrics and their potential relevance and impact for guiding more effective policymaking. Our index is particularly pertinent given that for the first time the EU has embarked on the creation of an EU-wide voluntary review which will assess the EU’s current progress towards SDG achievement and the potential for future progress [21]. We argue that the index presented in the paper is the most suitable tool that is currently available for such an assessment. Several factors contribute to the index’s suitability. For instance, we opt to use a relative, as opposed to an absolute, assessment of EU performance in so far as the performance of every EU country is calculated relative to the best and worst performers in EU for this indicator. As such, our index allows for the assessment of what can be reasonably expected within an EU context in terms of SDG performance. However, our index methodology is particularly advantageous to EU policymakers since it allows for the direct investigation of how variations in national policy relate to variations in national SDG performance.

Our index methodology presents several further significant innovations in sustainable development assessment and reporting that are particularly useful to EU policymakers. First, it is distinct in that it has been guided by an indicator-based, as opposed to a goal-based, approach avoiding the siloed reasoning that is antagonistic to SDG achievement, particularly for issues related to the environment [22]. An indicator-based approach is particularly beneficial to policymakers since it allows for areas of underperformance to be identified with a high degree of granularity thus enabling the development of tailored SDG policies. Second, it presents the first occasion that an innovative coding system (the classification of SDG targets as key Outcomes, Means-of-implementation (MoIs), or Linkages) has been applied in an EU context. The index’s unique indicator taxonomy, coupled with its indicator-based assessment, facilitates a nuanced approach to assessing performance in various dimensions of the SDGs while simultaneously allowing for micro and macro policy investigation. Third, by identifying the best and worst performers in the EU for each indicator, our index creates an opportunity for peer learning and competition which can significantly enhance the efficiency of SDG policy setting. For instance, it might be worthwhile for EU policymakers to investigate the practicality of adapting the national policies of the best-performing nation in a given indicator to an EU-wide context (i.e., via the introduction of EU legislation). As such, this index holds a unique position in the field of SDG reporting in that it has the capacity to transcend the domain of assessment and directly influence policy. Fourth, our index represents the most comprehensive assessment of SDG performance to date [2, 15, 16, 23–27]. Finally, the extensive database that has been compiled during the creation of this index holds significant promise for future national SDG reporting insofar as data relating to 166 unique SDG indicators has been collected for each country in the EU.

Annual time series national-level data related to 135 SDG targets that span the 17 SDGs were used to construct an index for 166 unique SDG indicators where the upper and lower bounds represent the measurement values of the best and worst in the EU class, respectively. Thus, the index score (0–1) represents the average distance between a nation and the best performer in the indicator in question. A unique taxonomy allows for indicators to be classified and aggregated into composite indices measuring performance in Outcome, MoI, and Linkage targets. Similarly, individual indicators were categorised into specific sustainable development dimensions and aggregated indices were created relating to social, economic, environmental, and governance dimensions of the SDGs. Finally, indicators were aggregated into one overall Composite Index that represents national performance in the SDGs as a whole.

## 2 Methodology

### 2.1 Indicator selection and classification

**Fig 1:**
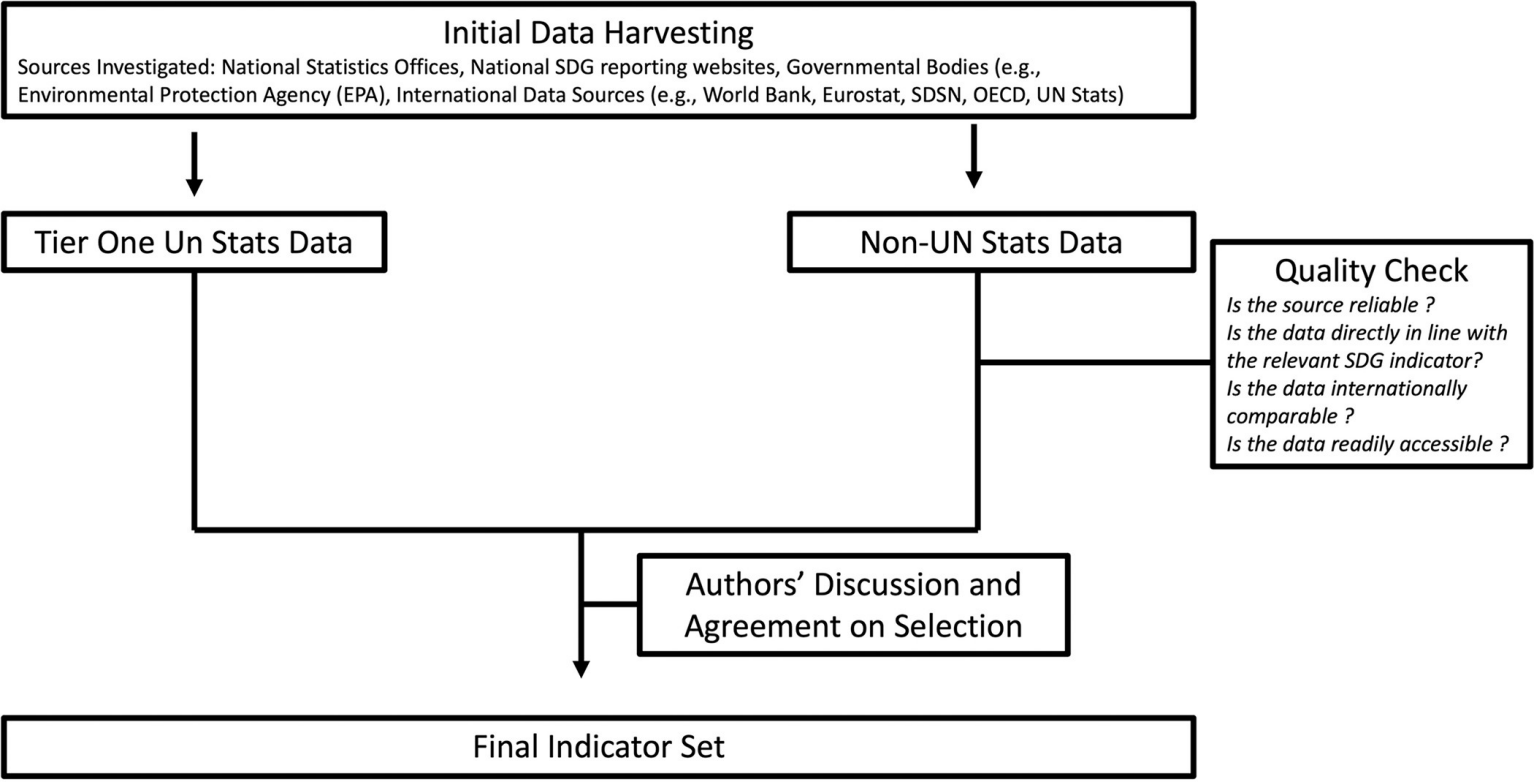
Schematic of the indicator selection process.

The standard approach to index construction involves the aggregation of normalised variables into a single composite index. In this context, it was necessary to first identify the indicators to be used in the creation of this index (Fig 1). In the initial stages, a search was undertaken to investigate potential data sources for each SDG indicator. Various sources of differing quality were found (e.g., national SDG reporting websites, national statistics systems (NSS)). It soon became apparent that the highest quality data could be derived from the United Nations Statistics Division (UNSD). The UNSD website contains open data on a multitude of SDG indicators. Various measures have been introduced to ensure the data is of the highest quality. An international organisation has been elected as the data custodian of each SDG indicator. It is the custodian agencies’ responsibility to ensure that data derived from NSS is harmonized and adheres to internationally agreed data standards. For example, the custodian organisation ensures that the data is open, transparent, is reported in a consistent standardised manner (via using Statistical Data and Metadata eXchanges (SDMX)). For these reasons, if data for an indicator was available from UNSD, this was taken as the best available source [28]. However, there are several indicators for which UNSD currently does not have data. In such instances, robust proxies that met specific data requirements that were created by the authors of this paper were used. The criteria were as follows: the identified data must be in direct alignment with the SDG indicator as per the official UN SDG framework, the source of the data must perform quality assurance procedures similar to those of the UN, the data must be readily available and accessible, and finally, the data must be internationally comparable The data for these indicators were derived from sources such as SDSN, Eurostat, and OECD (all data used is publicly available). Following the initial selection of indicators, several in-depth discussions took place between authors until a consensus regarding inclusion and exclusion was met.

As well as producing an overall composite index for each country in the EU, targets were classified as Outcome-based, Means of Implementation (MoI), and Linkage-based as well as those relating to the Social, Economic, Environmental, and Governance dimensions of the SDGs. Outcome-based indicators have been defined by the UN as indicators that refer to circumstances to be attained [29]. The UN defines MoI indicators, as those that measure SDG capacity building which might relate to technological development, resource mobilisation, inclusive globalised trade, or the generation of an enabling environment for SDG implementation. Means-of-implementation indicators are denoted by a letter in the SDG indicator framework, with the exception of SDG 17 in which all indicators are considered MoIs [29]. Finally, the authors of this paper introduced a third indicator class ‘Linkage-indicators’ that refer to those that span several goals. Linkage-indicators were also linguistically classified as indicators for which the corresponding target refers to more than one SDG pillar in the text. In adhering to the current state-of-the-art in SDG reporting and assessment [11], the authors aimed to classify indicators that might disproportionately accelerate overall SDG progress through the inclusion of a linkage-like indicator class.

Our previous research [30] presented a unique coding system that was used to identify each indicator in a proof of concept environmental SDG index for Ireland. The coding system is based on the taxonomy originally established during the UN Open Working Group (OWG) on SDG target development. More specifically, we take the implicit taxonomy used during the OWG-led SDG target development and explicitly add this taxonomy to the unique indicator code developed by UNSD. If an SDG target is assessed using only one indicator a ‘0’ is added to the UNSD code, if more than one indicator is used, a whole number starting from ‘1’ is serially added until all sub-indicators have been classified. A one-digit number is then added to illustrate that the indicator has been designated as an Outcome (‘1’), Linkage (‘2’), or an MOI (‘3’), by the OWG. While this coding system has previously been developed, this paper represents the first instance that it has been applied to an index that assesses the SDGs as a whole and that includes more than one EU nation.

Individual SDG targets were also aggregated into four SDG pillars: social, economic, environmental, and governance. In this sense, an index was developed for each SDG pillar. The Social Index consisted of indicators in SDGs 1–6, while the Economic Index contained indicators in SDGs 7–12. Indicators in SDGs 16–17 were used to populate the Governance Index, as defined by the UN [1]. Our previous work [31] developed a specific Environmental Index for Ireland and thus we were able to take a more nuanced approach to the construction of the Environmental Index in this study. Indeed, if all data were made available, the Environmental Index can contain 83 unique indicators spanning 16 of the 17 SDGs which have been described as environmentally-related by the United Nations Environment Programme [32]. This paper provides the first instance of an EU-wide SDG index being disaggregated in this manner. The entire list of indicators used in the composite index along with their classification status is shown in S1 Table in S1 File. It should be noted that there are several indicators for which there is no best or worst performer in the EU i.e., all EU nations perform the same. In such cases, our index returns a null value. Where available, annual data for each indicator for each country were collected for up to a maximum of ten years. As a consequence of its unique scope and alignment, our database holds significant potential for future national SDG performance assessment. The data collected is correct as of 01/09/2022. It should be noted that the data collection for this index was undertaken at a time when the United Kingdom (UK) was still part of the EU and thus the EU-28 is used rather than the current EU-27.

### 2.2 Weighting across indicators

Commonly, the aggregating of various indicators into an overall index requires a method of weighting that allows for subjective trade-offs that reflect the importance of particular indicators [33]. Indeed, both the Climate Change Performance Index and the Environmental Performance Index apply a differential weighting of indicators based on the subjective opinion of the relative importance of the indicators [34, 35]. However, such an unequal weighting system would contradict the indivisible nature of the SDGs while also introducing the risk of subjectivity bias [16]. Consequently, the norm in SDG index creation is to ascribe equal weight to each indicator in the overall composite index [2]. As a consequence of using an equally weighted approach, the ‘embedded weight’ of each SDG dimension is solely dependent on the number of indicators related to that dimension in proportion to the total number of indicators assessed. For example, our composite index contains 166 indicators of which 57 are directly related to the environment (i.e., environmental indicators account for 34% of the indicators used to create our index). One of the limitations of equal weighting is that poor performance in certain indicators tend to be smoothed out in a composite index value. Our index overcomes this limitation by presenting national performance in individual indicators and SDG dimensions, thus allowing for a highly accurate identification of a country’s areas of weak and strong performance.

### 2.3 Normalisation and aggregation

As can be expected with a complex framework like the SDGs, measurement values tend to vary significantly between indicators. For example, some measurement values relate to the percentage of children who are developmentally on track, while others relate to the amount of official development in millions of United States dollars. This high degree of variability necessitates a method of normalisation; individual data points need to be standardised between some upper and lower bounds so that they can be comparable on a single relative scale across indicators. Indices vary in their approach to normalisation. For example, in some cases, SDSN uses the absolute quantitative limits outlined in the SDG framework to guide upper and lower bounds e.g., full gender equality, a 50% reduction in the population in poverty, and universal access to clean water. Where no such quantitative threshold exists, normalisation values are set to either zero deprivation or universal access to indicators related to issues such as access to basic infrastructure (e.g., broadband access), public service coverage (e.g., health care coverage), and to those indicators that relate to the ’no-one-left-behind’ principle of the SDGs (e.g., equal educational opportunity). Otherwise, science-based thresholds, such as target values for CO2 per capita emissions, can be used. If the upper and lower bounds cannot be determined based on the previously-mentioned methods, the values of the top and bottom 2.5 percentile performers in an indicator are used [2].

With the exception of an index developed to assess the SDG performance of Italian cities [16], our index is unique in its approach to normalisation insofar as it is guided by peer performance. Specifically, in our index, the upper and lower bounds for an indicator are set to the values achieved by the best and worst performing countries, respectively, in the EU. One limitation associated with our approach is that the best-performing country in the EU might not be on track to achieve the SDG target and, as a consequence, the upper bound may not demonstrate SDG achievement. However, given the numerous policy implications of our approach (as discussed in this paper), we believe that our approach is to be favoured. Using our relative assessment approach, policymakers can examine the correlation between specific policies and SDG achievement. This allows for a tailored and targeted policymaking approach in specific areas. For instance, it would be beneficial for national policymakers to compare national policies to the policies of the best performing nations, particularly for those indicators for which their nation is underperforming. Further, our approach is beneficial in that it determines what can be reasonably expected from countries with relatively similar financial, political, and social ecosystems. It should be noted that there are indicators in our index for which various countries in the EU achieve the same maximum or minimum values in which case all such countries are considered to be top or bottom performers for this indicator.

Following data identification, an index score was calculated. There are certain indicators for which a higher measurement value represents poorer performance in the SDG indicator i.e., indicators such as SDG 9.4.1. ’CO2 emissions per unit of value added’. The outcomes related to such indicators are undesirable and thus these indicators were classified as ’undesirable’. In contrast, there are indicators for which the measurement values relate to desirable outcomes and thus were classified as ’desirable’ indicators. The following formulae (Eqs 1, 2, 3) were previously developed by the team and used to calculate Ireland’s score in an environmental SDG index [31]. In this study, Eqs 1 and 2 were used to standardise the data and place each EU country on a relative range:

$$I_{i}=\frac{\left({EU}_{i}-{Min}_{i}\right)}{\left({Max}_{i}-{Min}_{i}\right)}\;If\;the\;indicator\;target\;is\;desirable$$ [1]

$$I_{i}=1-\frac{\left({EU}_{i}-{Min}_{i}\right)}{\left({Max}_{i}-{Min}_{i}\right)}\;If\;the\;indicator\;target\;is\;undesirable$$ [2]

A composite SDG Index can be constructed using either an arithmetic, geometric, or harmonic mean. While each tendency measure was used during our analysis, the paper presents only the data relating to the arithmetic mean scores. There are several reasons why the arithmetic is preferred over the geometric or the harmonic mean. In general, the arithmetic mean tends to be norm in composite index construction since it is easily understood, and several analyses have found no significant advantage in using the geometric or the harmonic mean [36]. In the specific case of the SDGs, given that no EU nation performs well across the SDG indicator framework, the arithmetic mean is to be preferred. As shown in S3 Table in S1 File, all countries in the EU perform significantly poorer when the geometric and harmonic mean are used and, in several cases, the score approaches zero. However, as general SDG performance increases it may then be beneficial to consider using a ‘harsher’ mean for analysing country performance.

As shown in Eq 3, the arithmetic mean of our set of indicators is defined as the sum of the values of each observation divided by the total number of observations:

$$A=\frac{1}{n}\sum_{i=1}^{n}I_{i}=\frac{I_{1}+I_{2}+\cdots I_{n}}{n}$$ [3]

Eqs 4 and 5 were used to calculate the geometric and harmonic mean, respectively for each country in the EU (S3 Table in S1 File).


$$G=({\prod_{i=1}^{n}I_{i})}^{\frac{1}{N}}=\sqrt[{n}]{I_{1}I_{2}\ldots I_{n}}$$ [4]

$$H={\left(\frac{\sum_{i=1}^{n}I_{i}^{-1}}{n}\right)}^{-1}=\frac{n}{\frac{1}{I_{1}}+\frac{1}{I_{2}}+\cdots +\frac{1}{I_{n}}}$$ [5]

The result is an index that places each EU country on a scale relative to the best and worst performers in the EU for each indicator. The arithmetic mean score for all nations for which data was available was calculated for each indicator and used to determine the average EU performance.

### 2.4 Traffic light system

Our index takes a similar approach to data visualization as the SDSN SDG index in that a traffic light system is used [2]. Once the indicator scores have been normalised if the score is less than or equal to 0.33, the indicator is given a ‘red’ rating, if the score is between 0.33 and 0.66, the indicator is ‘orange’, and if the score is greater than or equal to 0.66, it is considered ‘green’.

### 2.5 GDP calculations

While gross domestic product (GDP) informs on the amount of wealth in a country, it does not consider a country’s capacity for wealth redistribution which is central to the SDG concept of leaving no one behind [1]. The influence of redistribution on SDG achievement is highlighted in the fact that countries with relatively high GDP per capita but poor wealth redistribution, such as the United States, tend to perform poorly in the SDGs [2]. Thus, we hypothesised that a combination of GDP per capita and some proxy for wealth redistribution (i.e., Gini coefficient) might better explain the results seen in our indices. In order to investigate the relationship between a country’s economic environment and its score in the various indices, regression analyses were run between a country’s GDPG (as shown in Eq 6 below) and a country’s score in the index in question.


$${GDPG=(GDP}_{per\;capita})(100-Gini\;Coefficient)$$ [6]

The higher the Gini Coefficient, the higher the inequality in a country. Since our index wanted to assess the relationship between a country’s index score and the country’s economic and redistributive capacity, using the Gini coefficient directly would penalise countries with low inequality/high wealth distribution. Consequently, 100 –Gini Coefficient was used. Regression analysis revealed that a greater correlation exists between GDP per capita and overall composite index score (R^2^ = 0.55) compared to GDPG (R^2^ = 0.44) and the Gini coefficient (R^2^ = 0.19). In theory, a better correlation for GDP compared to GDPG might appear surprising given that one of the primary aims of the SDGs is to reduce inequality [1]. However, these results are less surprising when one considers that the number of indicators that directly track GDP significantly outweigh those that directly relate to the Gini Coefficient. For instance, only one sub-indicator in our index (C10020122, used to track SDG 10.2.1. ‘proportion of people living below 50 per cent of median income, by sex, age and persons with disabilities’) assesses a nation’s performance in relation to the Gini Coefficient. By way of contrast, multiple indicators directly track GDP (e.g., SDG 8.1.1. ‘annual growth rate of real GDP per capita; SDG 8.2.1. ‘annual growth rate of real GDP per employed person; and SDG 17.3.1. ‘foreign direct investment’). Similarly, there are various indicators that are calculated using GDP as the denominator and thus, a higher GDP might shadow an otherwise poor performance (e.g., SDG 1.5.2. ‘direct economic loss attributed to disasters in relation to global GDP’; SDG 8.4.2. ‘domestic material consumption as a proportion of GDP’; SDG 9.4.1. ‘CO2 emissions per unit of GDP’).

Given that GDP appeared to be the indicator that best correlated with overall SDG performance, GDP per capita was used to investigate the relationship between a country’s financial context and its index score. It should be noted that due to the artificial inflation of the countries’ GDP, gross national income (GNI) and modified gross national income (GNI*) (a recently developed variation of GNI that appears to better account for foreign investment and thus more closely represents the economic strength of Irish residents compared to GDP and traditional GNI) were used for Luxembourg and Ireland, respectively [37]. The World Bank database was used as the source for GDP per capita and Gini Coefficient data (https://www.worldbank.org/en/home).

## 3 Results

### 3.1 Indicator-based EU assessment

**Fig 2:**
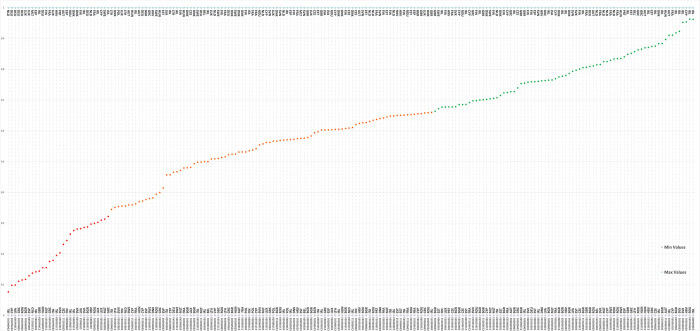
The average performance of the EU28 in each SDG indicator. The best and worst performers in each indicator are given in the top and bottom horizontal axes, respectively. The country codes used for all graphs are as follows: AUT; Austria, AVG; Average, BE; Belgium, BGR; Bulgaria, CYP; Cyprus, CZE; Czechia Republic, DEU; Germany, DNK; Denmark, ESP; Spain, EST; Estonia, FIN; Finland, FRA; France, GBR; United Kingdom, GRC; Greece, HRV; Croatia, HUN; Hungary, IRE; Ireland, ITA; Italy, LTU; Lithuania, LUX; Luxembourg, LVA; Latvia, MLT; Malta, NLD; Netherlands, POL; Poland, PRT; Portugal, ROU; Romania, SVK; Slovakia, SVN; Slovenia, SWE; Sweden.

Fig 2 depicts the average EU performance (i.e., the arithmetic mean score of all EU countries) in each indicator assessed along with the best and worst performers in class. As a country’s score tends towards 1, the closer that country is to being the best performer in the continent in each of the 166 indicators assessed. The average score varies significantly depending on the indicator in question. For example, the average performance for SDG 13.4.1. ‘Mobilized amount of United States dollars per year between 2020 and 2025 accountable towards the $100 billion commitment’ (essentially representing the financial commitments to the Paris Climate Agreement) is 0.099, indicating that the average country is 10% of the way towards the best performer in this indicator. This low average score suggests that most countries are significantly closer to the worst (i.e., Latvia) rather than the best performer (i.e., Germany). Similarly, on average, the continent performs poorly on SDG 14.8.1. ’Proportion of total research budget allocated to research in the field of marine technology’. With an average score of 0.144, nations tend to perform closer to the worst (i.e., Italy) rather than the best performer (i.e., Ireland). Our analysis reveals that MoI indicators represent a disproportionate number of indicators in the ’red’ traffic light class (i.e., a score less than or equal to 0.33). Despite making up approximately 27% of the total indicator set, MoIs represent approximately half of the indicators for which the average performance can be considered red. Despite this, there are also several indicators for which the EU average performance is high. For example, the average score for SDG 7.1.2. ’Proportion of population with primary reliance on clean fuels and technology’ is 0.963, suggesting that most nations are 96% of the way toward the best performer in this indicator. S2 Table in S1 File provides the rankings of all EU nations in each of the indicators assessed (along with the EU average score and variation between nations (i.e., standard deviation)) allowing for the high-level disaggregation of EU SDG performance.

### 3.2. Disaggregated SDG indices: Outcome, MoI, and linkage

**Fig 3:**
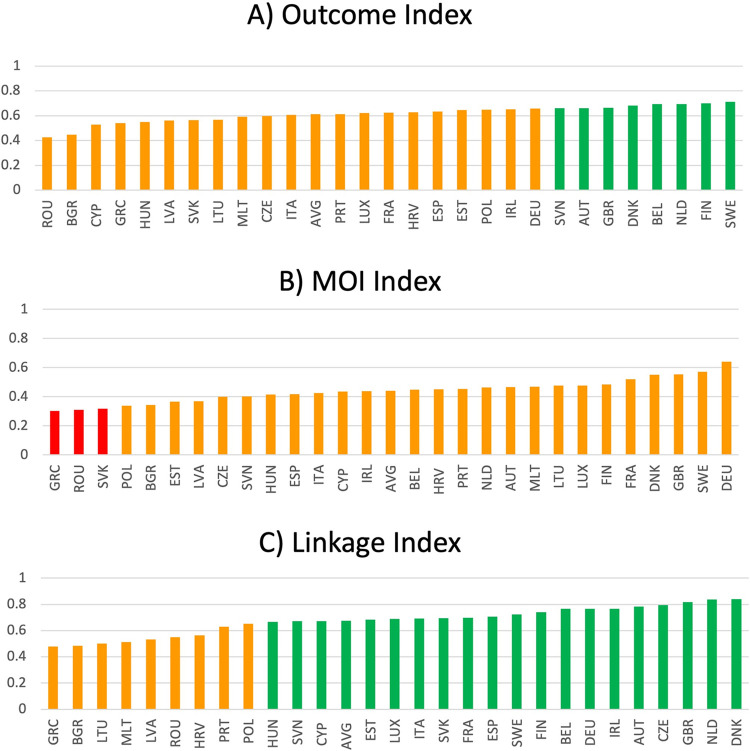
EU performance in Outcome (A), MoI (B), and Linkage (C) Indices.

Fig 3A–3C outlines the disaggregated Outcome, MoI, and Linkage indices. A total of 105, 45, and 16 unique SDG indicators were used to create each of the indices, respectively. The EU’s worst performance comes in the MoI Index i.e., no nation consistently performs close to the best performer for every MoI indicator. Indeed, the MoI Index is the only case where no country achieves a ’green’ (a score greater than or equal to 0.66) classification and several nations can be considered ‘red’. The high variability and poor average performance (average score = 0.44, range = 0.34) in the MoI Index might be partly explained by the nature of MoIs. Means-of-implementation indicators were recently developed to directly assess SDG capacity building [27]. Thus, the poor performance in the MoI Index might relate to the novelty of these measures and that optimum policy practices remain to be elucidated in Europe. In contrast, the better performance and lower variability in the Outcome Index (average score = 0.613, range = 0.29) might reflect that these indicators relate to preexisting efforts for which the best practices are already known. The Linkage Index score illustrates that, on average, nations are 68% of the way toward the best peformer in all Linkage indicators. The cross-disciplinary nature of linkage indicators might account for the high variability (range = 0.36) between the top and bottom performers. A proportion of linkage indicators are repeated throughout the index. As a consequence, if a linkage indicator is repeated twice in the index, a poor-performing nation is twice-penalised while a strong-performing country is twice-rewarded.

Our results reveal that countries that perform well in one index tend to perform well in other indices. However, the performance of several countries tends to be more inconsistent across indices. For example, Czechia is, on average, 79.4% towards the best performer in the Linkage Index but is significantly further from its best-performing peers in the MoI and Outcome indices, with scores of 0.399 and 0.596, respectively. Similarly, Portugal ranks as the 12^th^ best-performing country in the MoI Index (score = 0.541) but performs poorly in both the Linkage and Outcome indices, with 21^st^ ranking in both. Sweden is the top performer in the Outcome Index (score = 0.684) and second-best performer in the MoI Index (score = 0.572). However, Sweden performs significantly worse in the Linkage Index, ranking 10^th^ (score = 0.723). Thus, our results suggest that it might be prudent for Swedish policymakers to focus on national policies related to linkage indicators.

### 3.3. Disaggregated SDG indices: Social, economic, environmental, and governance pillars

**Fig 4:**
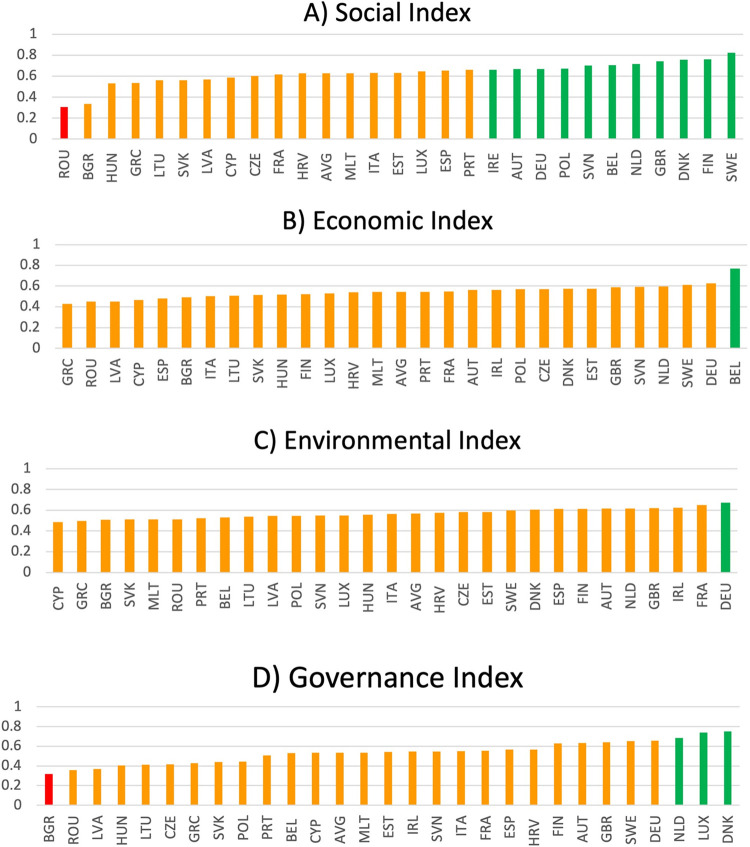
EU performance in the Social (A), Index (B), Environmental (C), and Governance (D) Indices.

Fig 4A–4D outlines the disaggregated Social, Economic, Environmental, and Governance indices. A total of 51, 36, 57, and 22 unique SDG indicators were used to create each of the indices, respectively. On average, the EU’s worst performance comes in the Governance Index (average score = 0.535, range = 0.43), illustrating that on average, nations are approximately 54% of the way toward the best performer in all Governance indicators assessed. The Social Index contains the highest score range and the highest average EU performance (average score = 0.627, range = 0.52). The pattern of performance in the Social Index might reflect the consistently poor relative performance of the bottom two nations (e.g., Romania and Bulgaria). Indeed, the third worst performing country in the Social Index (e.g., Hungary) achieves a score 0.2 points greater than Bulgaria. The continent achieves its least varied performance in the Environmental Index (average score = 0.568, range = 0.18). The low variability in this index might be explained by the fact that the EU generally sets a floor for environmental regulation and policy for the continent as a whole [38]. The Economic Index contains the second lowest, albeit still considerable, variation in index scores (average score = 0.543, range = 0.34).

Interestingly, Belgium is the best-performing country in the Economic Index (score = 0.768) and also performs well in the Social Index (score = 0.704, ranking = 6^th^). However, Belgium is among the worst-performing countries in the Environmental Index (score = 0.531, ranking = 21^st^). Therefore, it might be prudent for Belgian policymakers to thoroughly investigate Belgium’s current environmentally-related SDG policies. Germany is the only country whose performance warrants a ’green’ traffic light classification in the Environmental Index. Germany also performs well in the Economic (score = 0.625) and Governance (score = 0.659) indices ranking 2^nd^ and 4^th^, respectively. However, it comes in 9^th^ position in the Social Index with a score of 0.668, suggesting that it might be valuable for German policymakers to direct their focus to policies related to the social dimension of the SDGs.

### 3.4. Composite SDG index

**Fig 5:**
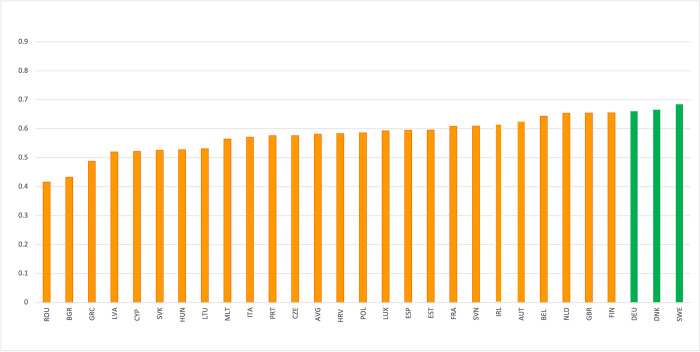
EU performance in the Composite SDG Index.

Fig 5 illustrates the results of the Composite Index. Sweden (score = 0.684), Denmark (score = 0.666), and Germany (score = 0.660) account for the top three performers and are the only nations categorised as ’green’. It is worth noting that these top three performers are still approximately 33% away from the top performer for the average SDG indicator, thus considerable work is still required for these countries. The benefits of our indicator-based approach is that we can specify with high granularity where improvement is needed in each country (S2 Table in S1 File). The EU average score of 0.581 illustrates that most nations achieve an ’orange’ index score (a score between 0.33–0.66) and are roughly 58% of the way toward the best performer in the SDGs as a whole. Romania (score = 0.416), Bulgaria (score = 0.433), and Greece (score = 0.490) make up the bottom three performers. It is worth noting that our results appear to reveal a pattern where Western European countries tend to perform better than Eastern European nations in our indices, which is consistent with other SDG assessments [2, 15]. A worse SDG performance might be expected in Eastern Europe given these nations typically have lower economic capability, higher inequality and poverty rates, and a worse environmental performance than Western Europe [2].

## 4 Discussion and conclusion

### 4.1. Understanding the European Union’s SDG performance

While it would be obtuse to attribute the entirety of a nation’s performance in such complex issues as the SDGs to a single factor, certain variables are known to significantly contribute to a country’s capacity for sustainable development. For example, various studies have found a relationship between a nation’s gross domestic product (GDP) and its potential for sustainable development [39]. A similar pattern is revealed in our index, where countries with high income per capita tend to perform better in the SDGs as a whole. Regression analysis revealed a correlation coefficient of 0.55 between a country’s GDP per capita (GDPC) and its Composite Index score. Therefore, while GDPC might at least, in part, explain a nation’s overall SDG performance, other factors are certainly involved. For example, despite a relatively low GDPC, Slovenia achieves a 10^th^ position ranking in the Composite Index. Its relatively robust performance might be explained by the strong legislative and political support that the SDGs receive in the country. For example, since 2017, the SDGs have been embedded in Slovenia’s National Development Strategy [40]. Similarly, national SDG reporting appears to be given appropriate consideration. Indeed, it is one of few countries to have submitted two separate Voluntary National Reviews to the UN [41, 42]. Furthermore, a government body (the Institute of Macroeconomic Analysis and Development) annually publishes national SDG performance reports [43]. Significant efforts have also been made to strengthen national policy coherence in Slovenia with one political body already established (and another planned) to work towards horizontal SDG policy coherence [44]. Overall, a nation’s engagement with the SDGs as well as its political and legislative ecosystem appear to be at least as important to SDG performance as its economic context. A nation’s economic capacity appears to have less of an influence on the performance in certain dimensions of the SDGs. For example, the correlation coefficient between a country’s Environmental Index score and GDPC was found to be 0.29. The fact that a moderate correlation between GDP and overall SDG performance persists despite the evidence that GDP tends to negatively impact environmental sustainability [45], might likely reflect the holistic definition of sustainable development according to the SDGs. According to the SDGs, sustainability relates to the economy, society, *and* the environment (i.e., we have previously described that the environmental pillar accounts for one third of the weight of the composite index). Similarly, as can be seen in the supplementary information, there are various environmentally-related indicators in the SDG indicator framework where a strong GDP might over shadow an otherwise poor performance (e.g., SDG 8.4.2. ‘domestic material consumption as a proportion of GDP).

### 4.2. The value of this index

By definition, the SDGs are a set of goals to be reached [1]. However, before a country may chart its course towards sustainable development, the distance between a country’s current starting point and SDG achievement must be evaluated. One of the primary aims of this study was to provide this information by presenting an accurate national-level assessment of the EU’s performance in 135 SDG targets as measured using 166 unique SDG indicators (see S2 Table in S1 File). In this sense, our index presents an opportunity to build upon the European Commission’s recently drafted first voluntary review of the progress towards, and current implementation of, the SDGs in the EU [46]. Given that the indicator set used to guide the development of the voluntary review can only assess 68 of the 231 SDG indicators, the EU currently risks missing 70% of the SDG indicator framework [26]. However, in this paper we present an more comprehensive data that assesses EU SDG achievement. For instance, through the indiscriminate use of all indicators for which sufficient data is currently available, our index avoids the subjectivity and potential bias introduced by selecting certain evaluation indicators over others [36, 47]. Furthermore, our index is significantly more comprehensive than any other in terms of the proportion of the SDG indicator framework evaluated [2, 15, 16, 23–27]. Indeed, the indices currently used to assess the EU’s SDG performance are only able to report on approximately half of the SDG indicator framework [2, 15, 26, 27]. Similarly, while the majority of indices use proxy data to evaluate performance, (as has been previously described in the methodology) the data used in this index is of the highest quality. The high quality of the data in our index enhances the translatability of our findings; EU policymakers can be confident that our results accurately reflect SDG performance. Overall, the presented index provides the most accurate and comprehensive assessment of SDG performance currently available.

Current SDG indices are largely restricted to the domain of assessment [2, 15, 27]. However, our index is unique in its practicality in that it can advise nations on the possible best path to SDG achievement. Given the complexity of the SDGs, it is unrealistic to expect a country to immediately develop robust and efficient policy measures for every SDG target, especially in countries that lack a strong history of robust sustainable development governance [47]. However, our use of a relative scale, as well as the identification of the best and worst performers in the EU, holds the potential to significantly catalyse the development of tailored SDG policy. In a similar fashion to the Belgian-specific policy analysis carried out by Bachus and colleagues [48], a poor-performing country can identify the top performer in a given indicator, analyse the relevant policies of this leader, and adopt such policies to fit their national context. For example, our analysis reveals that Ireland is one of the worst performers in SDG 2.5.1. ’ The proportion of local breeds with genetic material stored’. Therefore, it might be useful for Ireland to analyse the policies of the best performer (i.e., Spain) in the hope of adapting such policies to the Irish context. The peer policy learning environment enabled by our index can greatly enhance the efficiency of policy development [12, 49]; national policymakers can look to the best performers for direction rather than attempting to ’reinvent the wheel’. Thus, given that SDG achievement is underpinned by effective policy-making [13], this pan-EU Index holds the potential to significantly propel SDG achievement. Its capacity to transcend the domain of assessment and enter into the sphere of policy, makes our index the first of its kind in SDG reporting.

Our methodology reduces the complexity of understanding SDG performance and ultimately allow policymakers, scientists, alike to easily assess a country’s SDG performance in a variety of critical dimensions. While other indices have introduced some assessment of MoI indicators [23], our index is the first to use a taxonomy of Outcome, MoI, and Linkage indicators. As previously demonstrated in the case of an Irish Environmental Index [31], our taxonomy allows for a more nuanced approach to SDG assessment. For example, our results reveal that the EU, as a whole, requires significant improvement across the SDGs but specifically in MoIs. Given that MoIs directly relate to SDG capacity building [29], a poor performance does not bode well for the future sustainable development of the continent. Numerous bodies have recognised the necessity of transformative change for SDG achievement [2, 11]. While our criteria to classify Linkage indicators slightly diverges from that typically used to classify transformation indicators [11], the underlying principle is the same. Similar to transformations, a Linkage indicator represents a synergistic indicator wherein an improvement might disproportionately catalyse overall SDG achievement. As such, our results reveal that it might be prudent for countries that perform poorly in the Linkage Index, such as Greece and Malta, to initially focus on such indicators so as to accelerate overall SDG achievement. Future research might attempt to populate our Linkage Index with indicators that have been deemed highly interconnected by statistical methodologies such as that developed by Nilsson and colleagues [50].

To conclude, the index presented in this study represents the most comprehensive and accurate account of the EU’s sustainable development performance. Our results demonstrate that on average, EU nations are 58% of the way towards the best achiever in each of the indicators assessed. In creating this index, a database has been compiled containing data that directly aligns to 166 unique SDG indicators measured across several time points for each nation in the EU (approximately 8,000 data points). As a consequence, our database is unique in its scope and direct alignment to the UN SDG framework. By reducing the complexity of SDG assessment, our index can positively influence SDG awareness and understanding at various stakeholder levels. Similarly, the indices presented provide the information necessary to enhance effective decision-making at national and European levels. Our results reveal that SDG performance varies between nations and within nations depending on the indicator in question. Perhaps it is time for European and national-level policy development to reflect such heterogeneity and align with evidence such as that presented in this paper. The methods outlined are directly applicable to regions and countries outside the EU. Therefore, this assessment framework lays the foundation for the direct assessment of global SDG performance. Overall, our index provides a means by which the EU’s political agenda might be refocused, and the hope of SDG achievement reignited, thereafter.

## Supporting information

S1 File Supplementary tables. This file contains three tables: the meta-data for indicators used during index creation (S1), national indicator rankings (S2), the EU-composite index scores using arithmetic, geometric, and harmonic means (S3).
(DOCX)
