# Supplementary material for: Nation-based peer assessment of Europe’s Sustainable Development Goal performance
Source: PLoS One. 2023 Jun 28;18(6):e0287771. doi: 10.1371/journal.pone.0287771 (PMC10306182; doi:10.1371/journal.pone.0287771)
Supplement: S1 File — This file contains three tables: the meta-data for indicators used during index creation (S1), national indicator rankings (S2), the EU-composite index scores using arithmetic, geometric, and harmonic means (S3). (DOCX) [file pone.0287771.s001.docx]

Table S1 Meta-data for indicators used during index creation

| **Extended UNSD Indicator Codes** | **SDG Indicator** | **Data used in index** | **Dimension** | **Good (=)** | **Pillar** | **EUMin** | **EUMax** | **Source** | **Time point** |
| --- | --- | --- | --- | --- | --- | --- | --- | --- | --- |
| C01010101 | 1.1.1 Proportion of population below the international poverty line, by sex, age, employment status and geographical location (urban/rural) | Proportion of population below international poverty line, all age and both sex | Outcome | 0 | 1 | FRA | ROU | UN Stats | 2017 |
| C01020101 | 1.2.1 Proportion of population living below the national poverty line, by sex and age | Poverty rate after taxes and transfers, Poverty line 50%, unit= % of population. | Outcome | 0 | 1 | DNK | HUN | SDSN Index | 2021 |
| C01020201 | 1.2.2 Proportion of men, women and children of all ages living in poverty in all its dimensions according to national definitions | Proportion of population living in multidimensional poverty, both sex, all age | Outcome | 0 | 1 | CZE | BGR | UN Stats | 2019 |
| C01030101 | 1.3.1 Proportion of population covered by social protection floors/systems, by sex, distinguishing children, unemployed persons, older persons, persons with disabilities, pregnant women, newborns, work-injury victims and the poor and the vulnerable | Percentage of poor population receiving social assistance cash benefit | Outcome | 1 | 1 | BGR | IRE | UN Stats | 2016 |
| C01040111 | 1.4.1 Proportion of population living in households with access to basic services | Proportion of population using basic drinking water, ALL AREA | Outcome | 1 | 1 | IRE | AUT | UN Stats | 2020 |
| C01040121 | 1.4.1 Proportion of population living in households with access to basic services | Proportion of population using basic sanitation services, ALL Area | Outcome | 1 | 1 | ROU | AUT | UN Stats | 2020 |
| C01050102 | 1.5.1 Number of deaths, missing persons and directly affected persons attributed to disasters per 100,000 population | Number of deaths and missing persons attributed to disasters per 100,000 population | Linkage | 0 | 3 | CYP | FRA | UN Stats | 2017 |
| C01050202 | 1.5.2 Direct economic loss attributed to disasters in relation to global gross domestic product (GDP) | Direct economic loss attributed to disasters in relation to global gross domestic product (GDP) | Linkage | 0 | 3 | IRE | HRV | UN Stats | 2017 |
| C01050302 | 1.5.3 Number of countries that adopt and implement national disaster risk reduction strategies in line with the Sendai Framework for Disaster Risk Reduction 2015–2030 | Number of countries that adopt and implement national disaster risk reduction strategies in line with the Sendai Framework for Disaster Risk Reduction 2015–2030 | Linkage | 1 | 3 | IRE | GBR | UN Stats | 2018 |
| C01050402 | 1.5.4 Proportion of local governments that adopt and implement local disaster risk reduction strategies in line with national disaster risk reduction strategies | Proportion of local governments that adopt and implement local disaster risk reduction strategies in line with national disaster risk reduction strategies | Linkage | 1 | 3 | BGR | IRE | UN Stats | 2017 |
| C010a0103 | 1.a.1 Proportion of domestically generated resources allocated by the government directly to poverty reduction programmes | Development assistance grants for poverty by donor countries, % GNI | MOI | 1 | 1 | ITA | LUX | UN Stats | 2018 |
| C010a0203 | 1.a.2 Proportion of total government spending on essential services (education, health and social protection) | Proportion of government spending on education | MOI | 1 | 1 | ITA | SWE | UN Stats | 2017 |
| C02010101 | 2.1.1 Prevalence of undernourishment | Prevalence of undernourishment | Outcome | 0 | 1 | IRE | SVK | UN Stats | 2019 |
| C02010201 | 2.1.2 Prevalence of moderate or severe food insecurity in the population, based on the Food Insecurity Experience Scale (FIES) | Prevalence of severe food insecurity in the population | Outcome | 0 | 1 | SVN | ROU | UN Stats | 2019 |
| C02020101 | 2.2.1 Prevalence of stunting (height for age <-2 standard deviation from the median of the World Health Organization (WHO) Child Growth Standards) among children under 5 years of age | Prevalence of stunting (low-height-for-age) in children under 5 | Outcome | 0 | 1 | DEU | BGR | SDSN Index | 2021 |
| C02020301 | 2.2.3 Prevalence of anaemia in women aged 15 to 49 years, by pregnancy status (percentage) | Proportion of women aged 15-49 years with anaemia (%) | Outcome | 0 | 1 | LUX | BGR | Un Stats | 2019 |
| C02030111 | 2.3.1 Volume of production per labour unit by classes of farming/pastoral/forestry enterprise size | Productivity of small-scale food producers (agricultural output per labour day, PPP) (constant 2011 international $) | Outcome | 1 | 1 | HUN | AUT | UN Stats | 2016 |
| C02030121 | 2.3.1 Volume of production per labour unit by classes of farming/pastoral/forestry enterprise size | Productivity of large-scale food producers (agricultural output per labour day, PPP) (constant 2011 international $) | Outcome | 1 | 1 | GRE | ITA | UN Stats | 2016 |
| **Extended UNSD Indicator Codes** | **SDG Indicator** | **Data used in index** | **Dimension** | **Good (=)** | **Pillar** | **EUMin** | **EUMax** | **Source** | **Time point** |
| C02040112 | 2.4.1 Proportion of agricultural area under productive and sustainable agriculture | Cereal yield (tonnes/ hectare) | Linkage | 1 | 3 | CYP | NLD | SDSN Index | 2021 |
| C02040122 | 2.4.1 Proportion of agricultural area under productive and sustainable agriculture | Sustainable Nitrogen Management Index | Linkage | 0 | 3 | IRE | CYP | SDSN Index | 2021 |
| C02040132 | 2.4.1 Proportion of agricultural area under productive and sustainable agriculture | Yield gap closure (%) | Linkage | 1 | 3 | PRT | FRA | SDSN Index | 2021 |
| C02050101 | 2.5.1 Number of plant and animal genetic resources for food and agriculture secured in either medium- or long-term conservation facilities | Proportion of local breeds with genetic material stored | Outcome | 1 | 3 | IRE | ESP | UN Stats | 2021 |
| C02050301 | 2.5.2 Proportion of local breeds classified as being at risk, not at risk or at unknown level of risk of extinction | Proportion of local breeds classified as known being at risk | Outcome | 0 | 3 | HUN | CYP | UN stats | 2021 |
| C020a0103 | 2.a.1 The agriculture orientation index for government expenditures | The agriculture orientation index for government expenditures | MOI | 1 | 2 | ESP | LUX | UN Stats | 2018 |
| C020b0203 | 2.b.1 Agricultural export subsidies | Agricultural export subsidies (millions of current United States dollars) | MOI | 1 | 1 | ROU | CZE | UN Stats | 2004 |
| C020c0103 | 2.c.1 Indicator of food price anomalies | Consumer Price Index | MOI | 0 | 1 | LUX | POL | UN Stats | 2019 |
| C03010101 | 3.1.1 Maternal mortality ratio | Maternal mortality ratio | Outcome | 0 | 1 | GRE | LVA | UN Stats | 2017 |
| C03010201 | 3.1.2 Proportion of births attended by skilled health personnel | Proportion of births attended by skilled health personnel | Outcome | 1 | 1 | DNK | HRV | UN Stats | 2016 |
| C03020101 | 3.2.1 Under-5 mortality rate | Under-5 mortality rate | Outcome | 0 | 1 | SVN | ROU | UN Stats | 2019 |
| C03020201 | 3.2.2 Neonatal mortality rate | Neonatal mortality rate | Outcome | 0 | 1 | EST | MLT | UN Stats | 2019 |
| C03030101 | 3.3.1 Number of new HIV infections per 1,000 uninfected population, by sex, age and key populations | Number of new HIV infections per 1,000 uninfected population | Outcome | 0 | 1 | SVK | EST | UN Stats | 2018 |
| C03030201 | 3.3.2 Tuberculosis incidence per 100,000 population | Tuberculosis incidence per 100,000 population | Outcome | 0 | 1 | GRE | ROU | UN Stats | 2019 |
| C03030401 | 3.3.4 Hepatitis B incidence per 100,000 population | Hepatitis B incidence per 100,000 population | Outcome | 0 | 1 | IRE | FIN | UN Stats | 2015 |
| C03030501 | 3.3.5 Number of people requiring interventions against neglected tropical diseases | Number of people requiring interventions against neglected tropical diseases | Outcome | 0 | 1 | IRE | BGR | UN Stats | 2019 |
| C03040101 | 3.4.1 Mortality rate attributed to cardiovascular disease, cancer, diabetes or chronic respiratory disease | Mortality rate attributed to cardiovascular disease, cancer, diabetes or chronic respiratory disease | Outcome | 0 | 1 | CYP | BGR | UN Stats | 2019 |
| C03040201 | 3.4.2 Suicide mortality rate | Suicide mortality rate | Outcome | 0 | 1 | CYP | LTH | UN Stats | 2019 |
| C03050201 | 3.5.2 Harmful use of alcohol, defined according to the national context as alcohol per capita consumption (aged 15 years and older) within a calendar year in litres of pure alcohol | Alcohol per capita consumption (aged 15 years and older) within a calendar year in litres of pure alcohol | Outcome | 0 | 1 | ITA | CZE | UN Stats | 2019 |
| C03060101 | 3.6.1 Death rate due to road traffic injuries | Death rate due to road traffic injuries | Outcome | 0 | 1 | IRE | POL | UN Stats | 2019 |
| C03070201 | 3.7.2 Adolescent birth rate (aged 10–14 years; aged 15–19 years) per 1,000 women in that age group | Adolescent birth rate (aged 15–19 years) per 1,000 women in that age group | Outcome | 0 | 1 | DNK | BGR | UN Stats | 2018 |
| C03080101 | 3.8.1 Coverage of essential health services (defined as the average coverage of essential services based on tracer interventions that include reproductive, maternal, newborn and child health, infectious diseases, non-communicable diseases and service capacity and access, among the general and the most disadvantaged population) | Coverage of essential health services | Outcome | 1 | 1 | BGR | GBR | UN Stats | 2019 |
| C03080201 | 3.8.2 Proportion of population with large household expenditures on health as a share of total household expenditure or income | Proportion of population with large household expenditures (greater than 10%) on health as a share of total household expenditure or income | Outcome | 0 | 1 | FRA | CYP | UN Stats | 2010 |
| C03090101 | 3.9.1 Mortality rate attributed to household and ambient air pollution | Mortality rate attributed to household and ambient air pollution | Outcome | 0 | 3 | FIN | BGR | UN Stats | 2016 |
| **Extended UNSD Indicator Codes** | **SDG Indicator** | **Data used in index** | **Dimension** | **Good (=)** | **Pillar** | **EUMin** | **EUMax** | **Source** | **Time point** |
| C03090201 | 3.9.2 Mortality rate attributed to unsafe water, unsafe sanitation and lack of hygiene (exposure to unsafe Water, Sanitation and Hygiene for All (WASH) services) | Mortality rate attributed to unsafe water, unsafe sanitation and lack of hygiene (exposure to unsafe Water, Sanitation and Hygiene for All (WASH) services) | Outcome | 0 | 3 | FIN | DEU | UN Stats | 2016 |
| C03090301 | 3.9.3 Mortality rate attributed to unintentional poisoning | Mortality rate attributed to unintentional poisoning | Outcome | 0 | 3 | NLD | ROU | UN Stats | 2019 |
| C030a0103 | 3.a.1 Age-standardized prevalence of current tobacco use among persons aged 15 years and older | Age-standardized prevalence of current tobacco use among persons aged 15 years and older | MOI | 0 | 1 | DNK | BGR | UN Stats | 2018 |
| C030b0113 | 3.b.1 Proportion of the target population covered by all vaccines included in their national programme | Proportion of the target population with access to pneumococcal conjugate 3rd dose (PCV3) | MOI | 1 | 1 | AUT | HUN | UN Stats | 2019 |
| C030b0123 | 3.b.1 Proportion of the target population covered by all vaccines included in their national programme | Proportion of the target population with access to 3 doses of diphtheria-tetanus-pertussis (DTP3) | MOI | 1 | 1 | AUT | GRE | UN Stats | 2019 |
| C030b0133 | 3.b.1 Proportion of the target population covered by all vaccines included in their national programme | Proportion of the target population with access to affordable medicines and vaccines on a sustainable basis, human papillomavirus (HPV) | MOI | 1 | 1 | BGR | GBR | UN Stats | 2019 |
| C030b0143 | 3.b.1 Proportion of the target population covered by all vaccines included in their national programme | Proportion of the target population with access to measles-containing-vaccine second-dose (MCV2) | MOI | 1 | 1 | FRA | HUN | UN Stats | 2018 |
| C030c0103 | 3.c.1 Health worker density and distribution | Health worker density (dentistry, nursing, pharmacy, physiology) (per 10,000 population) | MOI | 1 | 1 | LVA | FIN | UN Stats | 2018 |
| C030d0103 | 3.d.1 International Health Regulations (IHR) capacity and health emergency preparedness | International Health Regulations (IHR) capacity; Average of the 13 SPAR core capacities | MOI | 1 | 1 | AUT | DNK | UN Stats | 2018 |
| C030d0213 | 3.d.2 Percentage of bloodstream infections due to selected antimicrobial-resistant organisms | Percentage of bloodstream infection due to methicillin-resistant Staphylococcus aureus (MRSA) among patients seeking care and whose blood sample is taken and tested (%) | MOI | 0 | 1 | NLD | CYP | UN Stats | 2019 |
| C030d0213 | 3.d.2 Percentage of bloodstream infections due to selected antimicrobial-resistant organisms | Percentage of bloodstream infection due to Escherichia coli resistant to 3rd-generation cephalosporin (e.g., ESBL- E. coli) among patients seeking care and whose blood sample is taken and tested (%) | MOI | 0 | 1 | NLD | ITA | UN Stats | 2019 |
| C04010111 | 4.1.1 Proportion of children and young people (a) in grades 2/3; (b) at the end of primary; and (c) at the end of lower secondary achieving at least a minimum proficiency level in (i) reading and (ii) mathematics, by sex | Proportion of children and young people achieving a minimum proficiency level in maths, lower secondary, both sex | Outcome | 1 | 1 | ROU | EST | UN Stats | 2018 |
| C04010121 | 4.1.1 Proportion of children and young people (a) in grades 2/3; (b) at the end of primary; and (c) at the end of lower secondary achieving at least a minimum proficiency level in (i) reading and (ii) mathematics, by sex | Proportion of children and young people achieving a minimum proficiency level in reading lower secondary, both sex | Outcome | 1 | 1 | BGR | EST | UN Stats | 2018 |
| C04010211 | 4.1.2. Completion rate (primary education, lower secondary education, upper secondary education) | Completion rate for lower secondary | Outcome | 1 | 1 | LUX | FIN | UN Stats | 2014 |
| C04010221 | 4.1.2. Completion rate (primary education, lower secondary education, upper secondary education) | Completion rate for upper secondary | Outcome | 1 | 1 | PRT | HRV | UN Stats | 2014 |
| C04020201 | 4.2.2 Participation rate in organized learning (one year before the official primary entry age), by sex | Participation rate in organized learning (one year before the official primary entry age), both sex | Outcome | 1 | 1 | BGR | FRA | UN Stats | 2018 |
| C04030101 | 4.3.1 Participation rate of youth and adults in formal and non-formal education and training in the previous 12 months, by sex | Participation rate of youth and adults in formal and non-formal education and training in the previous 12 months, both sex | Outcome | 1 | 1 | GRE | SWE | UN Stats | 2011 |
| **Extended UNSD Indicator Codes** | **SDG Indicator** | **Data used in index** | **Dimension** | **Good (=)** | **Pillar** | **EUMin** | **EUMax** | **Source** | **Time point** |
| C04040101 | 4.4.1 Proportion of youth and adults with information and communications technology (ICT) skills, by type of skill | Average of following skills: ARSP, CMFL,COPA, EPRS, PCPR, SOFT, TRAF, both sex | Outcome | 1 | 1 | IRE | NLD | UN Stats | 2017 |
| C04050111 | 4.5.1 Parity indices (female/male, rural/urban, bottom/top wealth quintile and others such as disability status, indigenous peoples and conflict-affected, as data become available) for all education indicators on this list that can be disaggregated | Average of gender parity indices for literacy and numeracy proficiency | Outcome | 1 | 1 | BGR | GBR | UN Stats | 2012 |
| C04050121 | 4.5.1 Parity indices (female/male, rural/urban, bottom/top wealth quintile and others such as disability status, indigenous peoples and conflict-affected, as data become available) for all education indicators on this list that can be disaggregated | Adjusted gender parity index for participation rate in formal and non-formal education and training | Outcome | 1 | 1 | NLD | LTH | UN Stats | 2011 |
| C04050131 | 4.5.1 Parity indices (female/male, rural/urban, bottom/top wealth quintile and others such as disability status, indigenous peoples and conflict-affected, as data become available) for all education indicators on this list that can be disaggregated | Gender parity index for youth/adults with information and communications technology (ICT) skills, by type of skill | Outcome | 1 | 1 | LUX | CYP | UN Stats | 2016 |
| C04050141 | 4.5.1 Parity indices (female/male, rural/urban, bottom/top wealth quintile and others such as disability status, indigenous peoples and conflict-affected, as data become available) for all education indicators on this list that can be disaggregated | Adjusted immigration status parity index for achieving at least a fixed level of proficiency, average of literacy and numeracy | Outcome | 1 | 1 | SWE | IRE | UN Stats | 2012 |
| C04050151 | 4.5.1 Parity indices (female/male, rural/urban, bottom/top wealth quintile and others such as disability status, indigenous peoples and conflict-affected, as data become available) for all education indicators on this list that can be disaggregated | Adjusted low to high socio-economic parity index for achieving a minimum proficiency level in reading and mathematics | Outcome | 1 | 1 | BGR | FIN | UN Stats | 2012 |
| C04050161 | 4.5.1 Parity indices (female/male, rural/urban, bottom/top wealth quintile and others such as disability status, indigenous peoples and conflict-affected, as data become available) for all education indicators on this list that can be disaggregated | Adjusted rural to urban parity index for achieving a minimum proficiency level in mathematics, lower secondary | Outcome | 1 | 1 | ROU | GBR | UN Stats | 2018 |
| C04050171 | 4.5.1 Parity indices (female/male, rural/urban, bottom/top wealth quintile and others such as disability status, indigenous peoples and conflict-affected, as data become available) for all education indicators on this list that can be disaggregated | Adjusted rural to urban parity index for achieving a minimum proficiency level in reading, lower secondary | Outcome | 1 | 1 | BGR | GBR | UN Stats | 2018 |
| C04060111 | 4.6.1 Proportion of population in a given age group achieving at least a fixed level of proficiency in functional (a) literacy and (b) numeracy skills, by sex | Proportion of population achieving at least a fixed level of proficiency in literacy skills, both sex, all age | Outcome | 1 | 1 | ITA | FIN | UN Stats | 2012 |
| C04060121 | 4.6.1 Proportion of population in a given age group achieving at least a fixed level of proficiency in functional (a) literacy and (b) numeracy skills, by sex | Proportion of population achieving at least a fixed level of proficiency in numeracy skills, both sex, all age | Outcome | 1 | 1 | ITA | FIN | UN Stats | 2012 |
| C04070111 | 4.7.1 Extent to which (i) global citizenship education and (ii) education for sustainable development, including gender equality and human rights, are mainstreamed at all levels in (a) national education policies; (b) curricula; (c) teacher education; and (d) student assessment | Extent to which global citizenship education and education for sustainable development are mainstreamed in national education policies | Outcome | 1 | 3 | GBR | FRA | UN Stats | 2020 |
| C04070121 | 4.7.1 Extent to which (i) global citizenship education and (ii) education for sustainable development, including gender equality and human rights, are mainstreamed at all levels in (a) national education policies; (b) curricula; (c) teacher education; and (d) student assessment | Extent to which global citizenship education and education for sustainable development are mainstreamed in curricula | Outcome | 1 | 3 | CZE | ROU | UN Stats | 2020 |
| **Extended UNSD Indicator Codes** | **SDG Indicator** | **Data used in index** | **Dimension** | **Good (=)** | **Pillar** | **EUMin** | **EUMax** | **Source** | **Time point** |
| C04070131 | 4.7.1 Extent to which (i) global citizenship education and (ii) education for sustainable development, including gender equality and human rights, are mainstreamed at all levels in (a) national education policies; (b) curricula; (c) teacher education; and (d) student assessment | Extent to which global citizenship education and education for sustainable development are mainstreamed in teacher education | Outcome | 1 | 3 | CZE | FRA | UN Stats | 2020 |
| C04070141 | 4.7.1 Extent to which (i) global citizenship education and (ii) education for sustainable development, including gender equality and human rights, are mainstreamed at all levels in (a) national education policies; (b) curricula; (c) teacher education; and (d) student assessment | Extent to which global citizenship education and education for sustainable development are mainstreamed in student assessment | Outcome | 1 | 3 | SVK | FRA | UN Stats | 2020 |
| C040a0113 | 4.a.1 Proportion of schools with access to (a) electricity; (b) the Internet for pedagogical purposes; (c) computers for pedagogical purposes; (d) adapted infrastructure and materials for students with disabilities; (e) basic drinking water; (f) single-sex basic sanitation facilities; and (g) basic handwashing facilities (as per the WASH indicator definitions) | Proportion of schools with access to adapted infrastructure and materials for students with disabilities | MoI | 1 | 1 | SVK | FRA | UN Stats | 2016 |
| C040a0123 | 4.a.1 Proportion of schools with access to (a) electricity; (b) the Internet for pedagogical purposes; (c) computers for pedagogical purposes; (d) adapted infrastructure and materials for students with disabilities; (e) basic drinking water; (f) single-sex basic sanitation facilities; and (g) basic handwashing facilities (as per the WASH indicator definitions) | Proportion of schools with access to basic drinking water, by education level | MoI | 1 | 1 | .. | .. | UN Stats | 2016 |
| C040a0133 | 4.a.1 Proportion of schools with access to (a) electricity; (b) the Internet for pedagogical purposes; (c) computers for pedagogical purposes; (d) adapted infrastructure and materials for students with disabilities; (e) basic drinking water; (f) single-sex basic sanitation facilities; and (g) basic handwashing facilities (as per the WASH indicator definitions) | Proportion of schools with access to the internet for pedagogical purposes | MoI | 1 | 1 | ITA | IRE | UN Stats | 2016 |
| C040a0143 | 4.a.1 Proportion of schools with access to (a) electricity; (b) the Internet for pedagogical purposes; (c) computers for pedagogical purposes; (d) adapted infrastructure and materials for students with disabilities; (e) basic drinking water; (f) single-sex basic sanitation facilities; and (g) basic handwashing facilities (as per the WASH indicator definitions) | Proportion of schools with access to computers for pedagogical purposes, | MoI | 1 | 1 | SVK | IRE | UN Stats | 2016 |
| C040a0153 | 4.a.1 Proportion of schools with access to (a) electricity; (b) the Internet for pedagogical purposes; (c) computers for pedagogical purposes; (d) adapted infrastructure and materials for students with disabilities; (e) basic drinking water; (f) single-sex basic sanitation facilities; and (g) basic handwashing facilities (as per the WASH indicator definitions) | Proportion of schools with access to electricity | MoI | 1 | 1 | .. | .. | UN Stats | 2016 |
| C040a0163 | 4.a.1 Proportion of schools with access to (a) electricity; (b) the Internet for pedagogical purposes; (c) computers for pedagogical purposes; (d) adapted infrastructure and materials for students with disabilities; (e) basic drinking water; (f) single-sex basic sanitation facilities; and (g) basic handwashing facilities (as per the WASH indicator definitions) | Proportion of schools with basic handwashing facilities | MoI | 1 | 1 | .. | .. | UN Stats | 2016 |
| C05010111 | 5.1.1 Whether or not legal frameworks are in place to promote, enforce and monitor equality and non-discrimination on the basis of sex | Legal frameworks that promote, enforce and monitor gender equality (percentage of achievement, 0 - 100) -- Area 1: overarching legal frameworks and public life | Outcome | 1 | 1 | FIN | ESP | UN Stats | 2020 |
| C05010121 | 5.1.1 Whether or not legal frameworks are in place to promote, enforce and monitor equality and non-discrimination on the basis of sex | Legal frameworks that promote, enforce and monitor gender equality (percentage of achievement, 0 - 100) -- Area 2: violence against women | Outcome | 1 | 1 | EST | ESP | UN Stats | 2020 |
| **Extended UNSD Indicator Codes** | **SDG Indicator** | **Data used in index** | **Dimension** | **Good (=)** | **Pillar** | **EUMin** | **EUMax** | **Source** | **Time point** |
| C05010131 | 5.1.1 Whether or not legal frameworks are in place to promote, enforce and monitor equality and non-discrimination on the basis of sex | Legal frameworks that promote, enforce and monitor gender equality (percentage of achievement, 0 - 100) -- Area 3: employment and economic benefits | Outcome | 1 | 1 | FRA | ESP | UN Stats | 2020 |
| C05010141 | 5.1.1 Whether or not legal frameworks are in place to promote, enforce and monitor equality and non-discrimination on the basis of sex | Legal frameworks that promote, enforce and monitor gender equality (percentage of achievement, 0 - 100) -- Area 4: marriage and family | Outcome | 1 | 1 | CYP | DEU | UN Stats | 2020 |
| C05020102 | 5.2.1 Proportion of ever-partnered women and girls aged 15 years and older subjected to physical, sexual or psychological violence by a current or former intimate partner in the previous 12 months, by form of violence and by age | Proportion of ever-partnered women and girls subjected to physical and/or sexual violence by a current or former intimate partner in the previous 12 months, age 15-49 | Linkage | 0 | 1 | ESP | FIN | UN Stats | 2018 |
| C05040111 | 5.4.1 Proportion of time spent on unpaid domestic and care work, by sex, age and location | Proportion of time spent on unpaid domestic and care work, female | Outcome | 0 | 1 | BEL | IRE | UN Stats | 2005 |
| C05040121 | 5.4.1 Proportion of time spent on unpaid domestic and care work, by sex, age and location | Proportion of time spent on unpaid domestic and care work, male | Outcome | 0 | 1 | IRE | BEL | UN Stats | 2005 |
| C05050102 | 5.5.1 Proportion of seats held by women in (a) national parliaments and (b) local governments | Proportion of seats held by women in national parliaments | Outcome | 1 | 1 | HUN | SWE | UN Stats | 2021 |
| C05050202 | 5.5.2 Proportion of women in managerial positions | Proportion of women in senior and middle managerial positions | Outcome | 1 | 1 | ITA | LVA | UN Stats | 2019 |
| C05060211 | 5.6.2 Number of countries with laws and regulations that guarantee full and equal access to women and men aged 15 years and older to sexual and reproductive health care, information and education | Extent to which countries have laws and regulations that guarantee full and equal access to women and men aged 15 years and older to sexual and reproductive health care, information and education: Section 1: Maternity Care (%) | Outcome | 1 | 1 | LVA | SWE | UN Stats | 2019 |
| C05060221 | 5.6.2 Number of countries with laws and regulations that guarantee full and equal access to women and men aged 15 years and older to sexual and reproductive health care, information and education | Extent to which countries have laws and regulations that guarantee full and equal access to women and men aged 15 years and older to sexual and reproductive health care, information and education: Section 2: Contraceptive and Family Planning (%) | Outcome | 1 | 1 | CZE | FIN | UN Stats | 2019 |
| C05060231 | 5.6.2 Number of countries with laws and regulations that guarantee full and equal access to women and men aged 15 years and older to sexual and reproductive health care, information and education | Extent to which countries have laws and regulations that guarantee full and equal access to women and men aged 15 years and older to sexual and reproductive health care, information and education: Section 3: Sexuality Education (%) | Outcome | 1 | 1 | GRE | DNK | UN Stats | 2019 |
| C05060241 | 5.6.2 Number of countries with laws and regulations that guarantee full and equal access to women and men aged 15 years and older to sexual and reproductive health care, information and education | Extent to which countries have laws and regulations that guarantee full and equal access to women and men aged 15 years and older to sexual and reproductive health care, information and education: Section 4: HIV and HPV (%) | Outcome | 1 | 1 | LTH | BEL | UN Stats | 2019 |
| C050b0103 | 5.b.1 Proportion of individuals who own a mobile telephone, by sex | Proportion of individuals who own a mobile telephone, both sex | MOI | 1 | 1 | FRA | FIN | UN Stats | 2018 |
| C050c0103 | 5.c.1 Proportion of countries with systems to track and make public allocations for gender equality and women’s empowerment | Proportion of countries with systems to track and make public allocations for gender equality and women's empowerment | MOI | 1 | 1 | CZE | AUT | UN Stats | 2021 |
| C06010101 | 6.1.1 Proportion of population using safely managed drinking water services | Proportion of population using safely managed drinking water services | Outcome | 1 | 3 | HUN | GRE | UN Stats | 2020 |
| C06020101 | 6.2.1 Proportion of population using (a) safely managed sanitation services and (b) a hand-washing facility with soap and water | Proportion of population using safely managed sanitation services | Outcome | 1 | 2 | HRV | AUT | UN Stats | 2020 |
| C06030101 | 6.3.1 Proportion of wastewater safely treated | Proportion of wastewater safely treated | Outcome | 1 | 3 | MLT | NLD | UN Stats | 2020 |
| C06030201 | 6.3.2 Proportion of bodies of water with good ambient water quality | Proportion of bodies of water with good ambient water quality (all bodies of water) | Outcome | 1 | 3 | GRE | FIN | UN Stats | 2020 |
| **Extended UNSD Indicator Codes** | **SDG Indicator** | **Data used in index** | **Dimension** | **Good (=)** | **Pillar** | **EUMin** | **EUMax** | **Source** | **Time point** |
| C06040101 | 6.4.1 Change in water-use efficiency over time | Water Use Efficiency (United States dollars per cubic meter) | Outcome | 1 | 3 | BGR | LUX | UN Stats | 2018 |
| C06040201 | 6.4.2 Level of water stress: freshwater withdrawal as a proportion of available freshwater resources | Level of water stress: freshwater withdrawal as a proportion of available freshwater resources | Outcome | 0 | 3 | LVA | MLT | UN Stats | 2018 |
| C06050101 | 6.5.1 Degree of integrated water resources management implementation (0–100) | Degree of integrated water resources management implementation (0–100) | Outcome | 1 | 3 | LTH | FRA | UN Stats | 2020 |
| C06050201 | 6.5.2 Proportion of transboundary basin area with an operational arrangement for water cooperation | Proportion of transboundary basin (rivers, lake basins, aquifiers) with an operational arrangement for water cooperation | Outcome | 1 | 3 | GBR | IRE | UN Stats | 2020 |
| C060a0103 | 6.a.1 Amount of water- and sanitation-related official development assistance that is part of a government-coordinated spending plan | Amount of water- and sanitation-related official development assistance that is part of a government-coordinated spending plan, current prices millions of United State Dollars (USD) | MOI | 1 | 3 | ROU | DEU | OECD | 2019 |
| C060b0113 | 6.b.1 Proportion of local administrative units with established and operational policies and procedures for participation of local communities in water and sanitation management | Countries with procedures in law/ policy for participation by... drinking water supply ( 10= Clearly Defined, 5= not clearly, 0 = NA | MOI | 1 | 3 | NLD | AUT | UN Stats | 2019 |
| 060b0123 | 6.b.1 Proportion of local administrative units with established and operational policies and procedures for participation of local communities in water and sanitation management | Countries with procedures in law/ policy for participation by... water resources and planning ( 10= Clearly Defined, 5= not clearly, 0 = NA) | MOI | 1 | 3 | .. | .. | UN Stats | 2019 |
| 060b0133 | 6.b.1 Proportion of local administrative units with established and operational policies and procedures for participation of local communities in water and sanitation management | Countries with users/communities participating in planning programs in rural drinking-water supply, by level of participation (3 = High; 2 = Moderate; 1 = Low; 0 = NA) | MOI | 1 | 3 | NLD | AUT | UN Stats | 2019 |
| 060b0143 | 6.b.1 Proportion of local administrative units with established and operational policies and procedures for participation of local communities in water and sanitation management | Countries with users/communities participating in planning programs in water resources planning and management, by level of participation (3 = High; 2 = Moderate; 1 = Low; 0 = NA) | MOI | 1 | 3 | HUN | NLD | UN Stats | 2019 |
| C07010101 | 7.1.1 Proportion of population with access to electricity | Proportion of population with access to electricity | Outcome | 1 | 2 | .. | .. | UN Stats | 2019 |
| C07010201 | 7.1.2 Proportion of population with primary reliance on clean fuels and technology | Proportion of population with primary reliance on clean fuels and technology | Outcome | 1 | 3 | ROU | IRE | UN Stats | 2018 |
| C07020101 | 7.2.1 Renewable energy share in the total final energy consumption | Renewable energy share in the total final energy consumption | Outcome | 1 | 3 | MLT | SWE | UN Stats | 2018 |
| C07030101 | 7.3.1 Energy intensity measured in terms of primary energy and GDP | Energy intensity measured in terms of primary energy and GDP (megajoules per constant 2011 purchasing power parity GDP) | Outcome | 0 | 3 | MLT | EST | UN Stats | 2018 |
| C070a0103 | 7.a.1 International financial flows to developing countries in support of clean energy research and development and renewable energy production, including in hybrid systems | International financial flows to developing countries in support of clean energy research and development and renewable energy production, including in hybrid systems (current prices USD millions) | MOI | 1 | 3 | CYP | DEU | OECD | 2018 |
| C070b013 | 7.b.1 Investments in energy efficiency as a proportion of GDP and the amount of foreign direct investment in financial transfer for infrastructure and technology to sustainable development services | Renewable energy public Research Development and Demonstration budget as a proportion of total energy public Research Development and Demonstration | MOI | 1 | 3 | SVK | IRE | OECD | 2020 |
| C070b023 | 7.b.1 Investments in energy efficiency as a proportion of GDP and the amount of foreign direct investment in financial transfer for infrastructure and technology to sustainable development services | Environmentally related official development assistance (ODA) as % of total ODA | MOI | 1 | 3 | ROU | FRA | OECD | 2019 |
| C08010101 | 8.1.1 Annual growth rate of real GDP per capita | Annual growth rate of real GDP per capita | Outcome | 1 | 2 | DEU | LTH | UN Stats | 2019 |
| C08020101 | 8.2.1 Annual growth rate of real GDP per employed person | Annual growth rate of real GDP per employed person | Outcome | 1 | 2 | ITA | LTH | UN Stats | 2019 |
| **Extended UNSD Indicator Codes** | **SDG Indicator** | **Data used in index** | **Dimension** | **Good (=)** | **Pillar** | **EUMin** | **EUMax** | **Source** | **Time point** |
| C20020312 | 8.4.2 Domestic material consumption, domestic material consumption per capita, and domestic material consumption per GDP | Domestic material consumption per capita | Linkage | 0 | 3 | ITA | EST | UN Stats | 2017 |
| C20020322 | 8.4.2 Domestic material consumption, domestic material consumption per capita, and domestic material consumption per GDP | Domestic material consumption per unit of GDP | Linkage | 0 | 3 | IRE | BGR | UN Stats | 2017 |
| C08050101 | 8.5.1 Average hourly earnings of female and male employees, by occupation, age and persons with disabilities | Average hourly earnings of all employees, Euro current prices | Outcome | 1 | 2 | LTH | DNK | UN Stats | 2018 |
| C08050201 | 8.5.2 Unemployment rate, by sex, age and persons with disabilities | Unemployment rate, Both sex, 15+ | Outcome | 0 | 2 | CZE | GRE | UN Stats | 2019 |
| C08060101 | 8.6.1 Proportion of youth (aged 15–24 years) not in education, employment or training | Proportion of youth (aged 15–24 years) not in education, employment or training | Outcome | 0 | 2 | NLD | ITA | UN Stats | 2019 |
| C08080111 | 8.8.1 Frequency rates of fatal and non-fatal occupational injuries, by sex and migrant status | Fatal occupational injuries per 100,000, Total | Outcome | 0 | 2 | NLD | ROU | UN Stats | 2015 |
| C08080121 | 8.8.1 Frequency rates of fatal and non-fatal occupational injuries, by sex and migrant status | Non-fatal occupational injuries per 100,000, Total | Outcome | 0 | 2 | ROU | FRA | UN Stats | 2015 |
| C08080201 | 8.8.2 Level of national compliance with labour rights (freedom of association and collective bargaining) based on International Labour Organization (ILO) textual sources and national legislation, by sex and migrant status | Level of national compliance with labour rights (freedom of association and collective bargaining) based on International Labour Organization (ILO) textual sources and national legislation, by sex and migrant status | Outcome | 1 | 2 | IRE | ROU | UN Stats | 2017 |
| C08090101 | 8.9.1 Tourism direct GDP as a proportion of total GDP and in growth rate | Tourism direct GDP as a proportion of total GDP (%) | Outcome | 1 | 2 | DNK | ESP | UN Stats | 2018 |
| C08100111 | 8.10.1 (a) Number of commercial bank branches per 100,000 adults and (b) number of automated teller machines (ATMs) per 100,000 adults | Number of automated teller machines (ATMs) per 100,000 adults | Outcome | 1 | 2 | SWE | AUT | UN Stats | 2019 |
| C08100121 | 8.10.1 (a) Number of commercial bank branches per 100,000 adults and (b) number of automated teller machines (ATMs) per 100,000 adults | Number of commercial bank branches per 100,000 adults | Outcome | 1 | 2 | FIN | LUX | UN Stats | 2019 |
| C08100201 | 8.10.2 Proportion of adults (15 years and older) with an account at a bank or other financial institution or with a mobile-money-service provider | Proportion of adults (15 years and older) with an account at a bank or other financial institution or with a mobile-money-service provider | Outcome | 1 | 2 | BGR | DNK | UN Stats | 2017 |
| C080a0103 | 8.a.1 Aid for Trade commitments and disbursements | Total official flows (commitments), USD Millions | MOI | 1 | 2 | GRE | DEU | UN Stats | 2019 |
| C09020101 | 9.2.1 Manufacturing value added as a proportion of GDP and per capita | Manufacturing value added as a proportion of GDP, constant 2015 USD | Outcome | 1 | 2 | LUX | IRE | UN Stats | 2020 |
| C09020201 | 9.2.2 Manufacturing employment as a proportion of total employment | Manufacturing employment as a proportion of total employment | Outcome | 1 | 2 | LUX | CZE | UN Stats | 2019 |
| C09030101 | 9.3.1 Proportion of small-scale industries in total industry value added | Proportion of small-scale industries in total industry value added | Outcome | 1 | 2 | LUX | CYP | UN Stats | 2018 |
| C09030201 | 9.3.2 Proportion of small-scale industries with a loan or line of credit | Proportion of small-scale industries with a loan or line of credit | Outcome | 0 | 2 | NLD | SVN | UN Stats | 2020 |
| C09040102 | 9.4.1 CO_2_ emission per unit of value added | CO2 emissions per Unit of GDP, Kg CO2 per constant GDP | Linkage | 0 | 3 | SWE | EST | UN Stats | 2018 |
| C09050101 | 9.5.1 Research and development expenditure as a proportion of GDP | Research and development expenditure as a proportion of GDP | Outcome | 1 | 2 | ROU | SWE | UN Stats | 2018 |
| C09050201 | 9.5.2 Researchers (in full-time equivalent) per million inhabitants | Researchers (in full-time equivalent) per million inhabitants | Outcome | 1 | 2 | ROU | DNK | UN Stats | 2018 |
| C090b0103 | 9.b.1 Proportion of medium and high-tech industry value added in total value added | Proportion of medium and high-tech industry value added in total value added | MOI | 1 | 2 | GRE | DEU | UN Stats | 2018 |
| C090c0103 | 9.c.1 Proportion of population covered by a mobile network, by technology | Proportion of population covered by a mobile network (4G) | MOI | 1 | 2 | IRE | DNK | UN Stats | 2019 |
| C10010101 | 10.1.1 Growth rates of household expenditure or income per capita among the bottom 40 per cent of the population and the total population | Growth rates of household expenditure or income per capita among the bottom 40 per cent of the population | Outcome | 1 | 2 | SVK | LUX | UN Stats | 2015 |
| **Extended UNSD Indicator Codes** | **SDG Indicator** | **Data used in index** | **Dimension** | **Good (=)** | **Pillar** | **EUMin** | **EUMax** | **Source** | **Time point** |
| C10020112 | 10.2.1 Proportion of people living below 50 per cent of median income, by sex, age and persons with disabilities | Gini coefficient adjusted for top income (1-100) | Linkage | 0 | 2 | SVN | ROU | SDSN Index | 2021 |
| C10020122 | 10.2.1 Proportion of people living below 50 per cent of median income, by sex, age and persons with disabilities | Palma Ratio | Linkage | 0 | 2 | SVK | BGR | SDSN Index | 2021 |
| C10020132 | 10.2.1 Proportion of people living below 50 per cent of median income, by sex, age and persons with disabilities | Elderly Poverty Ratio | Linkage | 0 | 2 | DNK | EST | SDSN Index | 2021 |
| C20020402 | 10.3.1 Proportion of population reporting having personally felt discriminated against or harassed in the previous 12 months on the basis of a ground of discrimination prohibited under international human rights law | Proportion of population reporting having personally felt discriminated against or harassed in the previous 12 months on the basis of a ground of discrimination prohibited under international human rights law | Linkage | 0 | 2 | IRE | FRA | UN Stats | 2019 |
| C10040101 | 10.4.1 Labour share of GDP, comprising wages and social protection transfers | Labour share of GDP, comprising wages and social protection transfers | Outcome | 1 | 2 | IRE | NLD | UN Stats | 2017 |
| C10050111 | 10.5.1 Financial Soundness Indicators | Net open position in foreign exchange to capital | Outcome | 1 | 2 | DNK | SVN | UN Stats | 2019 |
| C10050121 | 10.5.1 Financial Soundness Indicators | Non-performing loans net of provisions to capital | Outcome | 0 | 2 | HUN | GRE | UN Stats | 2019 |
| C10050131 | 10.5.1 Financial Soundness Indicators | Non-performing loans to total gross loans | Outcome | 0 | 2 | SWE | GRE | UN Stats | 2019 |
| C10050141 | 10.5.1 Financial Soundness Indicators | Regulatory Tier 1 capital to risk-weighted assests | Outcome | 1 | 2 | ESP | EST | UN Stats | 2019 |
| C10050151 | 10.5.1 Financial Soundness Indicators | Regulatory capital to assets | Outcome | 1 | 2 | DNK | IRE | UN Stats | 2019 |
| C10050161 | 10.5.1 Financial Soundness Indicators | Return on assets | Outcome | 1 | 2 | GRE | HUN | UN Stats | 2019 |
| C10070201 | 10.7.2 Number of countries with migration policies that facilitate orderly, safe, regular and responsible migration and mobility of people | Number of countries with migration policies that facilitate orderly, safe, regular and responsible migration and mobility of people | Outcome | 1 | 2 | IRE | ESP | UN Stats | 2019 |
| C10070301 | 10.7.3 Number of people who died or disappeared in the process of migration towards an international destinatio | Total deaths and disappearances recorded during migration (number) | Outcome | 0 | 2 | PRT | ESP | UN Stats | 2020 |
| C10070401 | 10.7.4 Proportion of the population who are refugees, by country of origin | Number of refugees per 100,000 population | Outcome | 0 | 2 | CYP | HRV | UN Stats | 2020 |
| C100a0103 | 10.a.1 Proportion of tariff lines applied to imports from least developed countries and developing countries with zero-tariff | Proportion of tariff lines applied to imports from least developed countries and developing countries with zero-tariff | MOI | 0 | 2 | FRA | HRV | UN Stats | 2019 |
| C100b0103 | 10.b.1 Total resource flows for development, by recipient and donor countries and type of flow (e.g. official development assistance, foreign direct investment and other flows) | Total resource flows for development by donor, millions USD | MOI | 1 | 2 | MLT | DEU | UN Stats | 2019 |
| C100c0103 | 10.c.1 Remittance costs as a proportion of the amount remitted | Average remittance costs of sending $200 for a sending country as a proportion of the amount remitted (% | MOI | 0 | 2 | EST | HUN | UN Stats | 2018 |
| C11010101 | 11.1.1 Proportion of urban population living in slums, informal settlements or inadequate housing | Proportion of urban population living in slums | Outcome | 0 | 2 | DNK | HUN | UN Stats | 2016 |
| C11020101 | 11.2.1 Proportion of population that has convenient access to public transport, by sex, age and persons with disabilities | Satisfaction with public transport | Outcome | 1 | 3 | LTH | LUX | SDSN Index | 2021 |
| C11050102 | 11.5.1 Number of deaths, missing persons and directly affected persons attributed to disasters per 100,000 population | Number of deaths and missing persons attributed to disasters per 100,000 population | Linkage | 0 | 3 | CYP | FRA | UN Stats | 2017 |
| **Extended UNSD Indicator Codes** | **SDG Indicator** | **Data used in index** | **Dimension** | **Good (=)** | **Pillar** | **EUMin** | **EUMax** | **Source** | **Time point** |
| C11050202 | 11.5.2 Direct economic loss in relation to global GDP, damage to critical infrastructure and number of disruptions to basic services, attributed to disasters | Direct economic loss attributed to disasters in relation to global gross domestic product (GDP) | Linkage | 0 | 3 | IRE | SVN | UN Stats | 2017 |
| C11060301 | 11.6.1 Proportion of urban solid waste regularly collected and with adequate final discharge out of total urban solid waste generated, by cities | Proportion of urban solid waste regularly collected and with adequate final discharge out of total urban solid waste generated, by cities | Outcome | 1 | 3 | HUN | AUT | UN Stats | 2015 |
| C11060201 | 11.6.2 Annual mean levels of fine particulate matter (e.g. PM2.5 and PM10) in cities (population weighted) | Annual mean levels of fine particulate matter (e.g. PM2.5 and PM10)All Area (population weighted) | Outcome | 0 | 3 | FIN | POL | UN Stats | 2016 |
| C110a01023 | 11.a.1 Proportion of population living in cities that implement urban and regional development plans integrating population projections and resource needs, by size of city | Countries that have national urban policies or regional development plans that respond to population dynamics; ensure balanced territorial development; and increase local fiscal space (1 = YES; 0 = NO) | Linkage | 1 | 2 | .. | .. | UN Stats | 2020 |
| C110b0102 | 11.b.1 Number of countries that adopt and implement national disaster risk reduction strategies in line with the Sendai Framework for Disaster Risk Reduction 2015–2030 | Number of countries that adopt and implement national disaster risk reduction strategies in line with the Sendai Framework for Disaster Risk Reduction 2015–2030 | Linkage | 1 | 3 | IRE | GBR | UN Stats | 2018 |
| C110b0202 | 11.b.2 Proportion of local governments that adopt and implement local disaster risk reduction strategies in line with national disaster risk reduction strategies | Proportion of local governments that adopt and implement local disaster risk reduction strategies in line with national disaster risk reduction strategies | Linkage | 1 | 3 | BGR | IRE | UN Stats | 2017 |
| C12010102 | 12.1.1 Number of countries with sustainable consumption and production (SCP) national action plans or SCP mainstreamed as a priority or a target into national policies | Countries with sustainable consumption and production (SCP) national action plans or SCP mainstreamed as a priority or target into national policies (1 = YES; 0 = NO) | Linkage | 1 | 3 | AUT | GBR | UN Stats | 2019 |
| C20020312 | 12.2.2 Domestic material consumption, domestic material consumption per capita, and domestic material consumption per GDP | Domestic Material Consumption per capita | Linkage | 0 | 3 | ITA | EST | UN Stats | 2017 |
| C20020322 | 12.2.2 Domestic material consumption, domestic material consumption per capita, and domestic material consumption per GDP | Domestic Material Consumption per unit of GDP | Linkage | 0 | 3 | IRE | BGR | UN Stats | 2017 |
| C12030101 | 12.3.1 (a) Food loss index and (b) food waste index | Food waste (KG of all domestic and commercial waste) per capita | Outcome | 0 | 3 | SVN | GRE | UN Stats | 2019 |
| C12040101 | 12.4.1 Number of parties to international multilateral environmental agreements on hazardous waste, and other chemicals that meet their commitments and obligations in transmitting information as required by each relevant agreement | The average of commitments met to Montreal Protocol, Rotterdam Convention, Basel Convention, and Stockholm Convention | Linkage | 1 | 3 | MLT | SWE | UN Stats | 2020 |
| C12040201 | 12.4.2 Hazardous waste generated per capita and proportion of hazardous waste treated, by type of treatment | Percentage of Hazardous Waste treated or disposed | Linkage | 1 | 3 | MLT | NLD | UN Stats | 2016 |
| C12050101 | 12.5.1 National recycling rate, tons of material recycled | National recycling rate of municipal waste | Outcome | 1 | 3 | MLT | DEU | Eurostat | 2018 |
| C12060101 | 12.6.1 Number of companies publishing sustainability reports | Number of companies publishing sustainability reports, Advanced Activity | Outcome | 1 | 3 | BGR | DEU | UN Stats | 2020 |
| **Extended UNSD Indicator Codes** | **SDG Indicator** | **Data used in index** | **Dimension** | **Good (=)** | **Pillar** | **EUMin** | **EUMax** | **Source** | **Time point** |
| C12070101 | 12.7.1 Number of countries implementing sustainable public procurement policies and action plans | Implementing sustainable public procurement policies and action plans | Outcome | 1 | 3 | Czech Republic | IRE | UN Stats | 2020 |
| C120b0103 | 12.b.1 Number of sustainable tourism strategies or policies and implemented action plans with agreed monitoring and evaluation tools | Implementation of standard accounting tools to monitor the economic and environmental aspects of tourism (number of tables) | MOI | 1 | 3 | IRE | DNK | UN Stats | 2019 |
| C120c0103 | 12.c.1 Amount of fossil-fuel subsidies per unit of GDP (production and consumption) and as a proportion of total national expenditure on fossil fuels | Fossil-fuel subsidies (consumption and production) as a proportion of total GDP (%) | MOI | 0 | 3 | LUX | BEL | UN Stats | 2019 |
| C13010102 | 13.1.1 Number of deaths, missing persons and directly affected persons attributed to disasters per 100,000 population | Number of deaths and missing persons attributed to disasters per 100,000 population | Linkage | 0 | 3 | CYP | FRA | UN Stats | 2017 |
| C13010202 | 13.1.2 Number of countries that adopt and implement national disaster risk reduction strategies in line with the Sendai Framework for Disaster Risk Reduction 2015–2030 | Number of countries that adopt and implement national disaster risk reduction strategies in line with the Sendai Framework for Disaster Risk Reduction 2015–2030 | Linkage | 1 | 3 | IRE | GBR | UN Stats | 2018 |
| C13010302 | 13.1.3 Proportion of local governments that adopt and implement local disaster risk reduction strategies in line with national disaster risk reduction strategies | Proportion of local governments that adopt and implement local disaster risk reduction strategies in line with national disaster risk reduction strategies | Linkage | 1 | 3 | BGR | IRE | UN Stats | 2017 |
| C13020201 | 13.2.2 Total greenhouse gas emissions per year | Total greenhouse gas emissions without LULUCF for Annex I Parties (Mt CO₂ equivalent) | Outcome | 0 | 2 | MLT | DEU | UN Stats | 2019 |
| C130a0103 | 13.a.1 Mobilized amount of United States dollars per year between 2020 and 2025 accountable towards the $100 billion commitment | Millions of Euro mobilized per year between 2020 and 2025 accountable towards the $100 billion commitment | MOI | 1 | 3 | LVA | DEU | Eurostat | 2019 |
| C14010111 | 14.1.1 Index of coastal eutrophication and floating plastic debris density | Percentage of chlorophyll-A deviations, remote sensing | Outcome | 0 | 3 | GRE | SVN | UN Stats | 2019 |
| C14010121 | 14.1.1 Index of coastal eutrophication and floating plastic debris density | Beach litter per square kilo | Outcome | 0 | 3 | IRE | BGR | UN Stats | 2019 |
| C14040101 | 14.4.1 Proportion of fish stocks within biologically sustainable levels | Fish caught from overexploited or collapsed stocks (% of total catch) | Outcome | 0 | 3 | EST | ITA | SDSN Index | 2021 |
| C14050101 | 14.5.1 Coverage of protected areas in relation to marine areas | Average proportion of Marine Key Biodiversity Areas (KBAs) covered by protected areas (%) | Outcome | 1 | 3 | CYP | BGR | UN Stats | 2020 |
| C14060101 | 14.6.1 Degree of implementation of international instruments aiming to combat illegal, unreported and unregulated fishing | Progress by countries in the degree of implementation of international instruments aiming to combat illegal, unreported and unregulated fishing (level of implementation: 1 lowest to 5 highest) | Outcome | 1 | 3 | .. | .. | UN Stats | 2020 |
| C14070102 | 14.7.1 Sustainable fisheries as a proportion of GDP in small island developing States, least developed countries and all countries | Sustainable fisheries as a proportion of GDP | Linkage | 1 | 3 | SVN | PRT | UN Stats | 2019 |
| C140a0103 | 14.a.1 Proportion of total research budget allocated to research in the field of marine technology | National ocean science expenditure as a share of total research and development funding (%) | MOI | 1 | 3 | GBR | IRE | UN Stats | 2017 |
| C140b0103 | 14.b.1 Degree of application of a legal/regulatory/ policy/institutional framework which recognizes and protects access rights for small-scale fisheries | Degree of application of a legal/regulatory/policy/institutional framework which recognizes and protects access rights for small-scale fisheries (level of implementation: 1 lowest to 5 highest) | MOI | 1 | 1 | .. | .. | UN Stats | 2020 |
| C140c0103 | 14.c.1 Number of countries making progress in ratifying, accepting and implementing through legal, policy and institutional frameworks, ocean-related instruments that implement international law, as reflected in the United Nations Convention on the Law of the Sea, for the conservation and sustainable use of the oceans and their resources | Score for the ratification of and accession to UNCLOS and its two implementing agreements (%) | MOI | 1 | 3 | EST | IRE | UN Stats | 2021 |
| C15010101 | 15.1.1 Forest area as a proportion of total land area | Forest area as a proportion of total land area | Outcome | 1 | 3 | MLT | FIN | UN Stats | 2020 |
| C15010211 | 15.1.2 Proportion of important sites for terrestrial and freshwater biodiversity that are covered by protected areas, by ecosystem type | Average proportion of Freshwater Key Biodiversity Areas (KBAs) covered by protected areas (%) | Outcome | 1 | 3 | CYP | DNK | UN Stats | 2020 |
| **Extended UNSD Indicator Codes** | **SDG Indicator** | **Data used in index** | **Dimension** | **Good (=)** | **Pillar** | **EUMin** | **EUMax** | **Source** | **Time point** |
| C15010221 | 15.1.2 Proportion of important sites for terrestrial and freshwater biodiversity that are covered by protected areas, by ecosystem type | Average proportion of Terrestrial Key Biodiversity Areas (KBAs) covered by protected areas | Outcome | 1 | 3 | ESP | LVA | UN Stats | 2020 |
| C15020111 | 15.2.1 Progress towards sustainable forest management | Annual forest area net change rate (%) | Outcome | 1 | 3 | NLD | MLT | UN Stats | 2020 |
| C15020121 | 15.2.1 Progress towards sustainable forest management | Proportion of forest area within legally established protected areas | Outcome | 1 | 3 | HRV | NLD | UN Stats | 2020 |
| C15020131 | 15.2.1 Progress towards sustainable forest management | Forest area certified under an independently verified certification scheme (thousands of hectares) | Outcome | 1 | 3 | MLT | FIN | UN Stats | 2019 |
| C15020141 | 15.2.1 Progress towards sustainable forest management | Proportion of forest area with long-term management plan | Outcome | 1 | 3 | MLT | FIN | UN Stats | 2020 |
| C15020151 | 15.2.1 Progress towards sustainable forest management | Change in Above ground biomass in forest, tonnes per hectare | Outcome | 1 | 3 | SVN | POL | UN Stats | 2020 |
| C15030101 | 15.3.1 Proportion of land that is degraded over total land area | Proportion of land that is degraded over total land area | Outcome | 0 | 3 | FIN | PRT | UN Stats | 2015 |
| C15040101 | 15.4.1 Coverage by protected areas of important sites for mountain biodiversity | Average proportion of Mountain Key Biodiversity Areas (KBAs) covered by protected areas (%) | Outcome | 1 | 3 | NLD | CZE | UN Stats | 2020 |
| C15040201 | 15.4.2. Mountain Green Cover Index | Mountain Green Cover Index | Outcome | 1 | 3 | SWE | HUN | UN Stats | 2018 |
| C15050101 | 15.5.1 Red List Index | Red List Index | Outcome | 0 | 3 | GRE | SWE | UN Stats | 2021 |
| C15060101 | 15.6.1 Number of countries that have adopted legislative, administrative and policy frameworks to ensure fair and equitable sharing of benefits | Average of adherence to the Nagoya Protocol, presence of legislative, administrative and policy framework or measures reported to the Access and Benefit-Sharing Clearing-House, presence of legislative, administrative and policy framework or measures reported through the Online Reporting System on Compliance of the International Treaty on Plant Genetic Resources for Food and Agriculture, and contract to the International Treaty on Plant Genetic Resources for Food and Agriculture | Outcome | 1 | 3 | IRE | DNK | UN Stats | 2018 |
| C15080101 | 15.8.1 Proportion of countries adopting relevant national legislation and adequately resourcing the prevention or control of invasive alien species | Average of two sub-indicators: Legislation, Regulation, Act related to the prevention of introduction and management of Invasive Alien Species (1 = YES, 0 = NO) and National Biodiversity Strategy and Action Plan (NBSAP) targets alignment to Aichi Biodiversity target 9 set out in the Strategic Plan for Biodiversity 2011-2020 (1 = YES, 0 = NO) | Outcome | 1 | 3 | CYP | Most EU Countries | UN Stats | 2016 |
| C15090201 | 15.9.1 Progress towards national targets established in accordance with Aichi Biodiversity Target 2 of the Strategic Plan for Biodiversity 2011–2020 | Average of two sub-indicators: Countries that established national targets in accordance with Aichi Biodiversity Target 2 of the Strategic Plan for Biodiversity 2011-2020 in their National Biodiversity Strategy and Action Plans (1 = YES; 0 = NO) and Countries with integrated biodiversity values into national accounting and reporting systems, defined as implementation of the System of Environmental-Economic Accounting (1 = YES; 0 = NO) | Outcome | 1 | 3 | .. | .. | UN Stats | 2020 |
| C150a0103 | 15.a.1 Official development assistance and public expenditure on conservation and sustainable use of biodiversity and ecosystems | Total official development assistance for biodiversity, by donor countries (millions of constant 2020 United States dollars) | MOI | 1 | 3 | GRE | FRA | UN Stats | 2018 |
| C150b0103 | 15.b.1 Official development assistance and public expenditure on conservation and sustainable use of biodiversity and ecosystems | Total official development assistance for biodiversity, by donor countries (millions of constant 2020 United States dollars) | MOI | 1 | 3 | GRE | FRA | UN Stats | 2018 |
| C16010101 | 16.1.1 Number of victims of intentional homicide per 100,000 population, by sex and age | Number of victims of intentional homicide per 100,000 population, both sex, all age | Outcome | 0 | 4 | SVN | LVA | UN Stats | 2018 |
| C16010311 | 16.1.3 Proportion of population subjected to (a) physical violence, (b) psychological violence and (c) sexual violence in the previous 12 months | Proportion of population subjected to physical violence in the previous 12 months | Outcome | 0 | 4 | IRE | FIN | SDSN Index | 2015 |
| C16010321 | 16.1.3 Proportion of population subjected to (a) physical violence, (b) psychological violence and (c) sexual violence in the previous 12 months | Proportion of population subjected to robbery violence in the previous 12 months | Outcome | 0 | 4 | NLD | CZE | SDSN Index | 2013 |
| **Extended UNSD Indicator Codes** | **SDG Indicator** | **Data used in index** | **Dimension** | **Good (=)** | **Pillar** | **EUMin** | **EUMax** | **Source** | **Time point** |
| C16010331 | 16.1.3 Proportion of population subjected to (a) physical violence, (b) psychological violence and (c) sexual violence in the previous 12 months | Proportion of population subjected to sexual violence in the previous 12 months | Outcome | 0 | 4 | FRA | GBR | SDSN Index | 2009 |
| C16010401 | 16.1.4 Proportion of population that feel safe walking alone around the area they live | Proportion of population that feel safe walking alone at night around the area they live | Outcome | 1 | 4 | BEL | SVN | UN Stats | 2021 |
| C16020201 | 16.2.2 Number of victims of human trafficking per 100,000 population, by sex, age and form of exploitation | Detected victims of human trafficking (Number), both sex, All age | Outcome | 0 | 4 | SWE | FRA | UN Stats | 2016 |
| C16030111 | 16.3.1 Proportion of victims of violence in the previous 12 months who reported their victimization to competent authorities or other officially recognized conflict resolution mechanisms | Police reporting for physical assault, both sex | Outcome | 1 | 4 | FRA | IRE | UN Stats | 2015 |
| C16030121 | 16.3.1 Proportion of victims of violence in the previous 12 months who reported their victimization to competent authorities or other officially recognized conflict resolution mechanisms | Police reporting for robbery, both sex | Outcome | 1 | 4 | FRA | DNK | UN Stats | 2014 |
| C16030131 | 16.3.1 Proportion of victims of violence in the previous 12 months who reported their victimization to competent authorities or other officially recognized conflict resolution mechanisms | Police reporting for sexual assault, both sex | Outcome | 1 | 4 | SWE | GBR | UN Stats | 2016 |
| C16030201 | 16.3.2 Unsentenced detainees as a proportion of overall prison population | Unsentenced detainees as a proportion of overall prison population (%) | Outcome | 0 | 4 | ROU | LUX | SDSN Index | 2017 |
| C16050101 | 16.5.1 Proportion of persons who had at least one contact with a public official and who paid a bribe to a public official, or were asked for a bribe by those public officials, during the previous 12 months | Corruption perception index | Outcome | 1 | 4 | BGR | DNK | SDSN Index | 2021 |
| C16050201 | 16.5.2 Proportion of businesses that had at least one contact with a public official and that paid a bribe to a public official, or were asked for a bribe by those public officials during the previous 12 months | Corruption perception index | Outcome | 1 | 4 | BGR | DNK | UN Stats | 2021 |
| C16090101 | 16.9.1 Proportion of children under 5 years of age whose births have been registered with a civil authority, by age | Proportion of children under 5 years of age whose births have been registered with a civil authority (% of children under 5 years of age) | Outcome | 1 | 4 | .. | .. | UN Stats | 2014 |
| C160a0103 | 16.a.1 Existence of independent national human rights institutions in compliance with the Paris Principles | Countries with National Human Rights Institutions in compliance with the Paris Principles, A status (1 = YES; 0 = NO) | MOI | 1 | 4 | .. | .. | UN Stats | 2018 |
| C17010103 | 17.1.1 Total government revenue as a proportion of GDP, by source | Total government revenue (budgetary central government) as a proportion of GDP (%) | MOI | 1 | 4 | IRE | DNK | UN Stats | 2019 |
| C17010203 | 17.1.2 Proportion of domestic budget funded by domestic taxes | Proportion of domestic budget funded by domestic taxes (% of GDP) | MOI | 1 | 4 | SVK | DNK | UN Stats | 2019 |
| C17020103 | 17.2.1 Net official development assistance, total and to least developed countries, as a proportion of the Organization for Economic Cooperation and Development (OECD) Development Assistance Committee donors’ gross national income (GNI) | Net official development assistance (ODA) as a percentage of OECD-DAC donors' GNI, by donor countries (%) | MOI | 1 | 4 | SVK | SWE | UN Stats | 2018 |
| C17030303 | 17.3.1 Foreign direct investment (FDI), official development assistance and South-South cooperation as a proportion of total domestic budget | Foreign direct investment (FDI) inflows (millions of US dollars) | MOI | 1 | 4 | IRE | GBR | UN Stats | 2018 |
| C17030203 | 17.3.2 Volume of remittances (in United States dollars) as a proportion of total GDP | Volume of remittances (in United States dollars) as a proportion of total GDP (%) | MOI | 1 | 4 | IRE | HRV | UN Stats | 2020 |
| C17060203 | 17.6.1. Fixed Internet broadband subscriptions per 100 inhabitants, by speed | Fixed Internet broadband subscriptions per 100 inhabitant (Any Speed) | MOI | 1 | 4 | POL | FRA | UN Stats | 2019 |
| **Extended UNSD Indicator Codes** | **SDG Indicator** | **Data used in index** | **Dimension** | **Good (=)** | **Pillar** | **EUMin** | **EUMax** | **Source** | **Time point** |
| C17080103 | 17.8.1 Proportion of individuals using the Internet | Internet users per 100 inhabitants | MOI | 1 | 4 | BGR | DNK | World Bank | 2018 |
| C17100103 | 17.10.1 Worldwide weighted tariff-average | Worldwide weighted tariff-average | MOI | 0 | 4 | .. | .. | UN Stats | 2017 |
| C17150113 | 17.15.1 Extent of use of country-owned results frameworks and planning tools by providers of development cooperation | Proportion of new development interventions drawn from country-led results framework- data from provider | MOI | 1 | 4 | ROU | FIN | UN Stats | 2017 |
| C17150123 | 17.15.1 Extent of use of country-owned results frameworks and planning tools by providers of development cooperation | Proportion of results indicators drawn from country-led results frameworks - data from provider | MOI | 1 | 4 | BEL | ROU | UN Stats | 2017 |
| C17150133 | 17.15.1 Extent of use of country-owned results frameworks and planning tools by providers of development cooperation | Proportion of results indicators which will be monitored using government sources and monitoring systems- data from provider | MOI | 1 | 4 | ROU | FIN | UN Stats | 2017 |
| C17160103 | 17.16.1 Number of countries reporting progress in multi-stakeholder development effectiveness monitoring frameworks that support the achievement of the sustainable development goals | Number of countries reporting progress in multi-stakeholder development effectiveness monitoring frameworks that support the achievement of the sustainable development goals, Provider (1 = YES; 0 = NO) | MOI | 1 | 4 | .. | .. | UN Stats | 2018 |
| C17180203 | 17.18.2 Number of countries that have national statistical legislation that complies with the Fundamental Principles of Official Statistics | Countries with national statistical legislation exists that complies with the Fundamental Principles of Official Statistics (1 = YES; 0 = NO) | MOI | 1 | 4 | .. | .. | UN Stats | 2018 |
| C17180303 | 17.18.3 Number of countries with a national statistical plan that is fully funded and under implementation, by source of funding | Number of countries with a national statistical plan that is fully funded and under implementation, by source of funding | MOI | 1 | 4 | .. | .. | UN Stats | 2018 |
| C17190203 | 17.19.2 Proportion of countries that (a) have conducted at least one population and housing census in the last 10 years; and (b) have achieved 100 per cent birth registration and 80 per cent death registration | Proportion of countries that (a) have conducted at least one population and housing census in the last 10 years; and (b) have achieved 100 per cent birth registration and 80 per cent death registration | MOI | 1 | 4 | .. | .. | UN Stats | 2016 |

Table S2 National indicator rankings

| **ID** | **Aut** | **Bel** | **Bgr** | **Hrv** | **Cyp** | **Cze** | **Dnk** | **Est** | **Fin** | **Fra** | **Deu** | **Grc** | **Hun** | **Ita** | **Ire** | **Lva** | **Lth** | **Lux** | **Mlt** | **Nld** | **Pol** | **Prt** | **Rou** | **Svk** | **Svn** | **Esp** | **Swe** | **Gbr** | **Average** | **St.Dev** |
| --- | --- | --- | --- | --- | --- | --- | --- | --- | --- | --- | --- | --- | --- | --- | --- | --- | --- | --- | --- | --- | --- | --- | --- | --- | --- | --- | --- | --- | --- | --- |
| C070a0103 | 12 | 8 | 26 | 24 | 28 | 17 | 7 | 22 | 11 | 3 | 1 | 19 | 16 | 6 | 10 | 27 | 25 | 13 | 23 | 5 | 14 | 15 | 18 | 20 | 21 | 9 | 4 | 2 | 0.10 | 0.22 |
| C13020201 | 8 | 11 | . | 25 | . | 15 | 9 | 22 | 10 | 2 | 1 | 21 | 18 | 7 | 12 | 26 | 19 | 13 | 24 | 6 | 14 | 20 | 23 | 16 | 17 | 4 | 5 | 3 | 0.10 | 0.25 |
| C08100111 | 11 | 7 | . | . | . | 15 | 6 | . | 10 | 2 | 1 | 19 | 14 | . | 12 | . | . | 9 | . | 4 | 13 | 16 | . | 17 | 18 | 8 | 5 | 3 | 0.11 | 0.26 |
| C060a0103 | 13 | 7 | . | 21 | . | 15 | 6 | . | 11 | 2 | 1 | . | 12 | 8 | 17 | . | 19 | 10 | . | 4 | 19 | 14 | 22 | 18 | 16 | 9 | 5 | 3 | 0.12 | 0.25 |
| C06040101 | 12 | 13 | 28 | 18 | 17 | 10 | 3 | 27 | 16 | 15 | 11 | 26 | 25 | 19 | 4 | 8 | 7 | 1 | 6 | 14 | 20 | 23 | 24 | 9 | 21 | 22 | 5 | 2 | 0.12 | 0.19 |
| C020a0103 | 8 | . | 4 | 14 | 9 | 6 | 17 | 7 | 10 | 23 | 5 | 26 | 21 | 24 | 3 | 20 | 11 | 1 | 2 | 25 | 12 | 19 | 15 | 22 | 13 | 27 | 18 | 16 | 0.13 | 0.19 |
| C15020111 | 15 | 24 | 9 | 14 | 25 | 16 | 3 | 7 | 17 | 6 | 21 | 22 | 19 | 5 | 2 | 13 | 12 | 22 | 1 | 28 | 11 | 10 | 4 | 18 | 27 | 20 | 26 | 8 | 0.14 | 0.19 |
| C02050101 | 4 | 8 | 10 | 14 | . | 6 | 14 | . | 12 | 2 | 14 | 14 | 14 | 5 | 14 | . | 14 | 14 | . | 3 | 12 | . | 7 | 14 | 10 | 1 | 8 | 14 | 0.14 | 0.23 |
| C04040101 | . | 2 | . | 4 | . | . | . | . | . | . | 1 | 3 | . | . | 5 | . | . | . | . | . | . | . | 5 | . | . | . | . | . | 0.14 | 0.20 |
| C140a0103 | . | . | 2 | . | . | . | . | . | 5 | 3 | 6 | . | . | 8 | 1 | . | . | . | . | 7 | 9 | . | . | . | . | 4 | . | 10 | 0.14 | 0.30 |
| C100b0103 | 11 | 9 | 24 | 22 | . | 17 | 10 | 25 | 12 | 3 | 1 | 16 | 6 | 8 | 13 | 26 | 23 | 15 | 27 | 2 | 14 | 21 | 18 | 20 | 19 | 5 | 7 | 4 | 0.15 | 0.25 |
| C15020131 | 22 | 16 | 12 | 7 | 23 | 9 | 19 | 10 | 1 | 3 | 4 | 23 | 17 | . | 15 | 8 | 13 | 20 | 23 | 20 | . | 14 | 5 | . | 18 | 6 | 2 | 11 | 0.16 | 0.27 |
| C150a0103 | 12 | 6 | . | . | . | 14 | 9 | . | 11 | 1 | 2 | 19 | . | 5 | 10 | . | . | 13 | . | 8 | 16 | 15 | . | 17 | 18 | 7 | 4 | 3 | 0.18 | 0.33 |
| C17030203 | 16 | 6 | 12 | 1 | 8 | 9 | 20 | 10 | 22 | 15 | 19 | 21 | 5 | 18 | 27 | 2 | 11 | 4 | . | 23 | 14 | 24 | 3 | 7 | 13 | 25 | 17 | 26 | 0.20 | 0.21 |
| C010a0103 | 11 | 7 | . | . | 11 | 17 | 4 | 17 | 7 | 11 | 6 | . | 7 | 17 | 7 | 17 | . | 1 | . | 3 | 17 | 11 | 17 | 17 | 16 | 11 | 2 | 4 | 0.21 | 0.26 |
| C050b0103 | 1 | 1 | . | . | 4 | 4 | . | . | 4 | . | 4 | 4 | . | 4 | . | . | 4 | 4 | . | 4 | . | 1 | . | . | . | 4 | . | . | 0.23 | 0.44 |
| C05010111 | 11 | . | 6 | 1 | 11 | 11 | 6 | 11 | 11 | 6 | 11 | 11 | 11 | . | 11 | 11 | 11 | 11 | 6 | 11 | 1 | 1 | 11 | 11 | 5 | 1 | 6 | 11 | 0.24 | 0.37 |
| C08080201 | 19 | 19 | 4 | 4 | 19 | 15 | 10 | 19 | 19 | 14 | 12 | 7 | 9 | 19 | 19 | 19 | 15 | 15 | 2 | 12 | 8 | 2 | 1 | 19 | 15 | 10 | 19 | 4 | 0.26 | 0.30 |
| C14070102 | . | 10 | 9 | 2 | . | . | 3 | . | . | . | . | . | . | . | 5 | . | 4 | . | 11 | . | 8 | 1 | 7 | . | 12 | . | 6 | . | 0.27 | 0.31 |
| C100b0103 | 7 | 20 | 10 | 9 | 10 | 2 | 1 | 10 | 4 | 20 | 10 | 20 | 10 | 2 | 20 | 10 | 4 | 20 | 10 | 7 | 20 | 20 | 20 | 10 | 10 | 19 | 4 | 20 | 0.28 | 0.30 |
| C17010203 | 16 | 12 | 8 | 15 | 6 | 22 | 1 | 17 | 13 | 23 | 19 | 14 | 18 | 9 | 3 | 25 | 11 | 4 | . | 7 | 21 | 10 | 27 | 26 | 24 | 20 | 2 | 5 | 0.28 | 0.23 |
| C020b0203 | . | . | 5 | . | 6 | 1 | . | . | . | . | . | . | 3 | . | . | . | . | . | . | . | 2 | . | 7 | 4 | . | . | . | . | 0.28 | 0.38 |
| C030b0123 | . | 4 | 13 | . | . | 11 | 6 | 18 | 1 | 5 | 2 | 15 | 17 | 15 | 3 | 19 | 10 | . | . | 7 | . | 12 | . | . | 9 | 14 | . | 8 | 0.28 | 0.21 |
| C070b013 | 8 | 14 | . | . | . | 11 | 2 | 3 | . | 9 | 5 | . | 12 | . | 1 | . | 13 | . | . | 6 | 7 | . | . | 15 | . | . | 4 | 10 | 0.29 | 0.26 |
| C15020121 | 8 | . | 13 | 22 | 17 | 21 | 19 | 10 | 15 | 7 | 6 | . | 9 | 23 | 12 | 14 | 4 | . | 16 | 1 | 3 | . | 2 | 5 | 11 | . | 20 | 18 | 0.30 | 0.24 |
| C070b023 | 11 | 7 | . | . | . | 15 | 2 | 21 | 14 | 1 | 3 | 23 | 16 | 6 | 12 | 22 | 17 | 9 | . | 5 | 20 | 19 | 24 | 8 | 18 | 13 | 4 | 10 | 0.30 | 0.22 |
| C09020101 | 9 | 17 | 19 | 15 | 27 | 2 | 12 | 14 | 11 | 24 | 4 | 23 | 6 | 13 | 1 | 20 | 7 | 28 | 26 | 21 | 10 | 18 | 8 | 5 | 3 | 22 | 16 | 25 | 0.31 | 0.20 |
| C09030101 | 25 | 24 | 15 | 7 | 1 | 14 | 21 | 6 | 19 | 17 | 27 | 8 | 22 | 2 | 26 | 12 | 20 | 28 | 4 | 11 | 18 | 3 | 23 | 13 | 5 | 10 | 15 | 9 | 0.31 | 0.20 |
| C07020101 | 5 | 26 | 12 | 7 | 22 | 19 | 4 | 8 | 2 | 18 | 16 | 13 | 20 | 15 | 25 | 3 | 6 | 17 | 27 | 27 | 23 | 9 | 10 | 21 | 11 | 14 | 1 | 24 | 0.31 | 0.26 |
| C08090101 | 7 | . | . | . | . | 9 | 13 | . | 11 | 3 | . | 4 | 4 | . | . | . | 9 | . | . | 8 | . | 2 | . | 11 | . | 1 | 6 | . | 0.31 | 0.29 |
| **ID** | **Aut** | **Bel** | **Bgr** | **Hrv** | **Cyp** | **Cze** | **Dnk** | **Est** | **Fin** | **Fra** | **Deu** | **Grc** | **Hun** | **Ita** | **Ire** | **Lva** | **Lth** | **Lux** | **Mlt** | **Nld** | **Pol** | **Prt** | **Rou** | **Svk** | **Svn** | **Esp** | **Swe** | **Gbr** | **Average** | **St.Dev** |
| C02030121 | 12 | 3 | 15 | 14 | 6 | 17 | 2 | 19 | 23 | 10 | 9 | 27 | 20 | 1 | 13 | 22 | 26 | 11 | 8 | 4 | 18 | 16 | 24 | 21 | 25 | 5 | 7 | . | 0.32 | 0.29 |
| C04050111 | 7 | 20 | 28 | 21 | 10 | 17 | 15 | 11 | 13 | 9 | 5 | 23 | 1 | 8 | 6 | 24 | 27 | 12 | 1 | 14 | 19 | 18 | 1 | 22 | 26 | 16 | 25 | 1 | 0.32 | 0.26 |
| C04050121 | 22 | 9 | 1 | 8 | 19 | 12 | 14 | 18 | 16 | 20 | 24 | 6 | 25 | 21 | 23 | 5 | 2 | 17 | 25 | 15 | 10 | 11 | 25 | 7 | 3 | 13 | 4 | 25 | 0.34 | 0.29 |
| C08020101 | 20 | 21 | 15 | 12 | 24 | 9 | 14 | 4 | 21 | 16 | 25 | 25 | 5 | 25 | 10 | 8 | 1 | 25 | 7 | 16 | 2 | 13 | 3 | 11 | 6 | 19 | 16 | 23 | 0.35 | 0.28 |
| C12060101 | . | . | . | . | . | . | . | . | 7 | . | 1 | . | . | 2 | 10 | . | . | 8 | . | 5 | . | 9 | 11 | . | . | 4 | 3 | 6 | 0.35 | 0.30 |
| C08050101 | 10 | 4 | 19 | . | 14 | 17 | 1 | 18 | . | 8 | 5 | 16 | 22 | 11 | 3 | 19 | 22 | 2 | 13 | 8 | . | 22 | 21 | . | 15 | 11 | 7 | 6 | 0.35 | 0.30 |
| C15020151 | 16 | 22 | 1 | 12 | 13 | 10 | 21 | 27 | 22 | 8 | 3 | 22 | 9 | 22 | 4 | 15 | 11 | 5 | . | 14 | 1 | 22 | 6 | 20 | 17 | 19 | 18 | 7 | 0.35 | 0.30 |
| C08100121 | 21 | 8 | 2 | 10 | 6 | 17 | 18 | 26 | 27 | 7 | 22 | 19 | 15 | 4 | 16 | 24 | 23 | 1 | 11 | 25 | 9 | 5 | 14 | 13 | 12 | 3 | 20 | . | 0.36 | 0.25 |
| C17020103 | . | . | . | . | . | . | . | . | 4 | 3 | 2 | 5 | . | . | . | . | . | . | . | . | . | . | . | 6 | . | . | 1 | . | 0.36 | 0.38 |
| C08100111 | 1 | 12 | 9 | 3 | 25 | 21 | 23 | 15 | 27 | 8 | 4 | 17 | 19 | 10 | 13 | 20 | 26 | 5 | 22 | 24 | 14 | 2 | 16 | 18 | 11 | 7 | 28 | 6 | 0.36 | 0.28 |
| C02050301 | 19 | 22 | 8 | 5 | 25 | 10 | 10 | 25 | 9 | 7 | 13 | 3 | 1 | 18 | 5 | 17 | 14 | 24 | . | 20 | 10 | 2 | . | 14 | 22 | 4 | 21 | 14 | 0.36 | 0.28 |
| C16030111 | . | . | . | . | . | . | . | . | . | 3 | . | . | . | . | 1 | . | . | . | . | . | . | . | . | . | . | . | 2 | . | 0.36 | 0.55 |
| C02030111 | 1 | 3 | 26 | 11 | 17 | 21 | 6 | 18 | 5 | 19 | 4 | 13 | 27 | 2 | 22 | 25 | 14 | 7 | 12 | 15 | 23 | 24 | 16 | 10 | 20 | 8 | 9 | . | 0.37 | 0.27 |
| C05010141 | 18 | . | 18 | 9 | 21 | 21 | 5 | 9 | 21 | 7 | 1 | 21 | 9 | . | 9 | 9 | 21 | 21 | 9 | 5 | 2 | 2 | 18 | 9 | 9 | 9 | 7 | 2 | 0.37 | 0.27 |
| C15050101 | 9 | 21 | 13 | 10 | 27 | 17 | 19 | 23 | 26 | 2 | 20 | 1 | 5 | 8 | 11 | 24 | 25 | 22 | 7 | 14 | 18 | 4 | 6 | 15 | 12 | 3 | 28 | 16 | 0.38 | 0.35 |
| C10050151 | . | 20 | 4 | 3 | 8 | 15 | 24 | 2 | . | 23 | 19 | 5 | . | 17 | 1 | 9 | 21 | 13 | 10 | 22 | 7 | 11 | . | 14 | 6 | 12 | 18 | 16 | 0.38 | 0.28 |
| C09050101 | . | 4 | . | 20 | 25 | 9 | 3 | 12 | 5 | 6 | 2 | 18 | 11 | 13 | 19 | 24 | 21 | 17 | 23 | 7 | 16 | 14 | 26 | 22 | 8 | 15 | 1 | 10 | 0.39 | 0.29 |
| C10050161 | . | 11 | 4 | 2 | 24 | 5 | 19 | 5 | . | 21 | 23 | 25 | 1 | 22 | 13 | 16 | 7 | 16 | 10 | 15 | 14 | 12 | . | 8 | 3 | 18 | 9 | 20 | 0.40 | 0.26 |
| C10050141 | . | 16 | 9 | 4 | 12 | 10 | 8 | 1 | . | 20 | 18 | 21 | 21 | 24 | 3 | 6 | 2 | 5 | 15 | 11 | 19 | 23 | . | 17 | 14 | 25 | 7 | 13 | 0.41 | 0.26 |
| C16010331 | . | . | . | . | . | . | . | 3 | . | 1 | . | . | . | . | . | . | . | . | . | 2 | . | . | . | . | . | . | . | . | 0.46 | 0.51 |
| C05050202 | 15 | 16 | 5 | 26 | 27 | 21 | 23 | 21 | 8 | 11 | 20 | 18 | 9 | 28 | 17 | 1 | 6 | 24 | 19 | 25 | 3 | 7 | 12 | 13 | 4 | 14 | 2 | 10 | 0.46 | 0.29 |
| C02040122 | 5 | 21 | 13 | 8 | 28 | 10 | 3 | 18 | 19 | 4 | 12 | 20 | 2 | 16 | 1 | 14 | 9 | 23 | 26 | 24 | 17 | 27 | 7 | 6 | 22 | 25 | 10 | 15 | 0.46 | 0.21 |
| C08010101 | 24 | 20 | 5 | 9 | 7 | 13 | 18 | 10 | 23 | 21 | 28 | 16 | 5 | 27 | 8 | 11 | 1 | 22 | 2 | 19 | 4 | 14 | 3 | 15 | 12 | 17 | 26 | 25 | 0.47 | 0.29 |
| C04050141 | 10 | . | . | . | 3 | 5 | 12 | 6 | 14 | 15 | 11 | . | . | 7 | 1 | . | 4 | . | . | 13 | . | . | . | 2 | . | 9 | 16 | 8 | 0.47 | 0.32 |
| C15010101 | 7 | 6 | 11 | 14 | 23 | 13 | 24 | 4 | 1 | 18 | 16 | 20 | 22 | 17 | 26 | 5 | 12 | 15 | 28 | 27 | 19 | 10 | 21 | 8 | 3 | 9 | 2 | 25 | 0.47 | 0.24 |
| C16050101 | 8 | 8 | 28 | 25 | 17 | 20 | 1 | 10 | 2 | 12 | 5 | 23 | 26 | 21 | 11 | 17 | 15 | 5 | 21 | 4 | 19 | 14 | 26 | 24 | 15 | 13 | 2 | 7 | 0.48 | 0.31 |
| C09020201 | 13 | 18 | 8 | 11 | 27 | 1 | 21 | 10 | 15 | 19 | 6 | 24 | 4 | 9 | 22 | 16 | 13 | 28 | 20 | 26 | 5 | 12 | 6 | 3 | 2 | 17 | 23 | 25 | 0.48 | 0.25 |
| C16030131 | . | . | . | . | . | . | . | . | . | 1 | . | . | . | . | . | . | . | . | . | . | . | . | . | . | . | . | 2 | . | 0.48 | 0.68 |
| C030b0143 | 24 | 23 | 22 | 4 | 19 | 24 | 15 | 19 | 11 | 26 | 11 | 26 | 1 | 17 | 7 | 7 | 13 | 15 | 4 | 17 | 13 | 3 | . | 2 | 7 | 7 | 4 | 19 | 0.49 | 0.28 |
| C16050201 | 8 | 9 | 28 | 24 | 16 | 16 | 1 | 10 | 2 | 12 | 6 | 27 | 26 | 22 | 10 | 19 | 16 | 5 | 21 | 4 | 14 | 13 | 25 | 23 | 14 | 19 | 2 | 6 | 0.49 | 0.31 |
| C010a0203 | 17 | 10 | 7 | 25 | 2 | 22 | 3 | 8 | 12 | 23 | 16 | 28 | 21 | 27 | 5 | 11 | 13 | 26 | 6 | 9 | 15 | 14 | 20 | 24 | 18 | 19 | 1 | 4 | 0.50 | 0.26 |
| C12050101 | 3 | 5 | 20 | 23 | 26 | 19 | 7 | 22 | 13 | 11 | 1 | 25 | 15 | 8 | 14 | 24 | 6 | 9 | 28 | 4 | 18 | 21 | 27 | 16 | 2 | 17 | 10 | 12 | 0.50 | 0.26 |
| **ID** | **Aut** | **Bel** | **Bgr** | **Hrv** | **Cyp** | **Cze** | **Dnk** | **Est** | **Fin** | **Fra** | **Deu** | **Grc** | **Hun** | **Ita** | **Ire** | **Lva** | **Lth** | **Lux** | **Mlt** | **Nld** | **Pol** | **Prt** | **Rou** | **Svk** | **Svn** | **Esp** | **Swe** | **Gbr** | **Average** | **St.Dev** |
| C20020402 | . | . | . | . | . | . | . | . | . | 2 | . | . | . | . | 1 | . | . | . | . | . | . | . | . | . | . | . | . | . | 0.50 | 0.71 |
| C05040111 | . | 1 | . | . | . | . | . | . | . | . | . | . | . | . | 2 | . | . | . | . | . | . | . | . | . | . | . | . | . | 0.50 | 0.71 |
| C05040121 | . | 2 | . | . | . | . | . | . | . | . | . | . | . | . | 1 | . | . | . | . | . | . | . | . | . | . | . | . | . | 0.50 | 0.71 |
| C11020101 | 5 | 14 | 23 | 25 | 27 | 3 | 9 | 9 | 16 | 17 | 7 | 20 | 14 | 26 | 19 | 11 | 28 | 1 | 11 | 2 | 24 | 22 | 21 | 17 | 8 | 11 | 4 | 6 | 0.51 | 0.26 |
| C02040112 | 9 | 2 | 13 | 4 | 28 | 16 | 17 | 27 | 26 | 5 | 8 | 21 | 7 | 15 | 3 | 25 | 24 | 12 | 18 | 1 | 23 | 19 | 11 | 14 | 10 | 20 | 22 | 6 | 0.51 | 0.26 |
| C04030101 | 9 | 14 | 23 | . | 11 | 16 | 4 | 8 | 5 | 6 | 7 | 26 | 13 | 20 | 24 | 21 | 22 | 2 | 18 | 3 | 25 | 10 | 27 | 12 | 17 | 14 | 1 | 19 | 0.51 | 0.24 |
| C17150133 | 5 | 8 | . | . | . | . | 7 | . | 1 | 4 | 6 | . | . | 2 | 3 | . | . | . | . | . | . | 9 | 10 | . | . | . | . | . | 0.51 | 0.27 |
| C06050101 | 5 | 13 | 23 | 6 | 3 | 15 | 2 | 12 | 15 | 1 | 7 | 9 | 20 | 18 | 14 | . | 24 | . | 9 | 4 | 21 | 22 | 18 | 24 | 24 | 8 | 9 | 17 | 0.52 | 0.27 |
| C090b0103 | 13 | 9 | 20 | 24 | 19 | 8 | 2 | 22 | 12 | 6 | 1 | 28 | 4 | 15 | 3 | 26 | 23 | 27 | 18 | 5 | 21 | 25 | 11 | 10 | 17 | 16 | 7 | 14 | 0.52 | 0.28 |
| C030b0113 | 18 | 9 | 27 | 24 | 25 | 21 | 1 | 19 | 3 | 23 | 15 | 27 | 20 | 6 | 8 | 25 | 12 | 4 | 10 | 6 | 11 | 13 | 16 | 22 | 5 | 13 | 17 | 2 | 0.52 | 0.29 |
| C05050102 | 6 | 4 | 19 | 14 | 25 | 21 | 7 | 17 | 2 | 8 | 13 | 24 | 28 | 9 | 23 | 15 | 18 | 12 | 27 | 11 | 16 | 5 | 26 | 22 | 20 | 3 | 1 | 10 | 0.52 | 0.27 |
| C17150123 | 2 | 10 | . | . | . | . | 9 | . | 3 | 7 | 4 | . | . | 5 | 6 | . | . | . | . | . | . | 8 | 1 | . | . | . | . | . | 0.53 | 0.29 |
| C030b0133 | . | 12 | 24 | 18 | 17 | 23 | 1 | 16 | 2 | 11 | 5 | . | 22 | 8 | 21 | 15 | 7 | 14 | 25 | 6 | . | 13 | 19 | 20 | 10 | 9 | 4 | 3 | 0.53 | 0.27 |
| C03050201 | 16 | 10 | 22 | 3 | 11 | 28 | 6 | 9 | 8 | 19 | 26 | 7 | 13 | 1 | 24 | 27 | 25 | 21 | 2 | 5 | 15 | 17 | 20 | 12 | 18 | 23 | 4 | 14 | 0.53 | 0.22 |
| C16030121 | . | . | . | . | . | . | 1 | . | . | 4 | . | . | . | . | 2 | . | . | . | . | . | . | . | . | . | . | . | 3 | . | 0.53 | 0.42 |
| C11060201 | 14 | 16 | 27 | 25 | 24 | 19 | 7 | 3 | 1 | 11 | 12 | 22 | 21 | 20 | 5 | 15 | 10 | 8 | 17 | 13 | 28 | 4 | 18 | 26 | 23 | 6 | 2 | 9 | 0.53 | 0.27 |
| C17060203 | 23 | 7 | 21 | 24 | 10 | 12 | 2 | 15 | 16 | 1 | 4 | 5 | 14 | 20 | 18 | 26 | 22 | 11 | . | 3 | 27 | 9 | 25 | 19 | 17 | 13 | 6 | 8 | 0.54 | 0.26 |
| C03080201 | . | 14 | 16 | 4 | 19 | 3 | 5 | . | 9 | 1 | 2 | 17 | 11 | 12 | 10 | . | 13 | 6 | 18 | . | . | 19 | 15 | 7 | . | 8 | . | . | 0.54 | 0.36 |
| C12030101 | . | . | . | . | . | . | . | . | 3 | 8 | 4 | 9 | . | . | 6 | . | 2 | . | 7 | . | . | . | 5 | . | 1 | . | . | . | 0.55 | 0.29 |
| C16030201 | 5 | 9 | . | . | . | . | 10 | . | 4 | 7 | 6 | 8 | . | . | 3 | . | . | 11 | . | . | . | . | 1 | . | . | 2 | . | . | 0.55 | 0.27 |
| C12040201 | 20 | 12 | 3 | 18 | 6 | 17 | 4 | 2 | 7 | 11 | 5 | . | 8 | 19 | 25 | 24 | 21 | 26 | 27 | 1 | 9 | 14 | 16 | 22 | 13 | 10 | 15 | 23 | 0.56 | 0.28 |
| C04050131 | 12 | 9 | 3 | 1 | 11 | 13 | 22 | 24 | 26 | 16 | 4 | 25 | 7 | 6 | 14 | 27 | 28 | 10 | 5 | 1 | 20 | 18 | 15 | 17 | 21 | 8 | 19 | 23 | 0.56 | 0.22 |
| C020c0103 | 2 | 3 | 24 | 4 | 8 | 12 | 18 | 17 | 15 | 26 | 5 | 7 | 22 | 13 | 11 | 10 | 19 | 1 | 9 | 27 | 28 | 16 | 25 | 23 | 6 | 20 | 20 | 14 | 0.57 | 0.25 |
| C17080103 | 10 | 9 | 28 | 23 | 13 | 17 | 1 | 7 | 8 | 15 | 6 | 26 | 22 | 25 | 12 | 14 | 20 | 2 | 16 | 4 | 21 | 24 | 27 | 18 | 19 | 11 | 5 | 3 | 0.57 | 0.25 |
| C10010101 | . | 10 | . | . | . | . | 9 | . | 6 | 6 | 10 | . | . | 10 | 1 | . | . | 1 | . | 4 | . | 1 | . | 13 | . | . | 6 | 4 | 0.57 | 0.18 |
| C10020112 | 9 | 5 | 23 | 18 | 14 | 8 | 2 | 16 | 3 | 11 | 12 | 27 | 17 | 21 | 10 | 22 | 26 | 15 | 6 | 4 | 25 | 24 | 28 | 13 | 1 | 20 | 7 | 19 | 0.57 | 0.30 |
| C17030303 | 14 | 6 | 22 | 21 | 25 | 10 | 20 | 18 | 26 | 5 | 2 | 15 | 12 | 8 | 28 | 24 | 23 | 27 | 16 | 3 | 9 | 11 | 13 | 17 | 19 | 4 | 7 | 1 | 0.57 | 0.19 |
| C05060211 | . | . | . | . | . | 6 | 5 | . | 2 | . | 9 | 10 | . | . | . | 11 | 4 | . | 8 | 2 | . | . | . | . | . | . | 1 | 7 | 0.57 | 0.33 |
| C10070201 | 1 | 1 | 13 | . | . | 1 | 13 | 1 | 1 | 1 | 13 | 1 | . | 13 | 13 | 13 | 1 | . | 13 | . | . | 1 | 13 | . | 13 | 1 | 1 | 1 | 0.57 | 0.51 |
| C100a0103 | 15 | 4 | 21 | 28 | 24 | 13 | 11 | 27 | 12 | 1 | 7 | 20 | 18 | 5 | 17 | 23 | 16 | 22 | 26 | 8 | 9 | 3 | 14 | 19 | 25 | 6 | 10 | 2 | 0.57 | 0.26 |
| C07030101 | 12 | 22 | 26 | 1 | 10 | 24 | 4 | 28 | 27 | 18 | 13 | 14 | 19 | 7 | 3 | 17 | 16 | 5 | 2 | 15 | 21 | 9 | 8 | 24 | 20 | 11 | 23 | 6 | 0.58 | 0.28 |
| C03060101 | 12 | 16 | 26 | 21 | 16 | 18 | 4 | 11 | 6 | 13 | 5 | 25 | 20 | 15 | 1 | 22 | 22 | 9 | 9 | 8 | 27 | 24 | 27 | 19 | 13 | 6 | 1 | 1 | 0.58 | 0.33 |
| **ID** | **Aut** | **Bel** | **Bgr** | **Hrv** | **Cyp** | **Cze** | **Dnk** | **Est** | **Fin** | **Fra** | **Deu** | **Grc** | **Hun** | **Ita** | **Ire** | **Lva** | **Lth** | **Lux** | **Mlt** | **Nld** | **Pol** | **Prt** | **Rou** | **Svk** | **Svn** | **Esp** | **Swe** | **Gbr** | **Average** | **St.Dev** |
| C05020102 | 9 | 16 | 22 | 10 | 2 | 12 | 4 | 13 | 27 | 17 | . | 18 | 21 | 7 | 4 | 24 | 20 | 7 | 10 | 18 | 2 | 15 | 26 | 22 | 6 | 1 | 24 | 13 | 0.58 | 0.22 |
| C16010401 | 5 | 28 | 24 | 3 | 20 | 19 | 6 | 11 | 2 | 17 | 14 | 23 | 25 | 18 | 16 | 26 | 20 | 3 | 12 | 7 | 20 | 7 | 26 | 14 | 1 | 9 | 10 | 13 | 0.58 | 0.27 |
| C03080101 | 14 | 7 | 26 | 23 | 17 | 18 | 7 | 18 | 11 | 9 | 4 | 18 | 23 | 11 | 11 | . | 26 | 2 | 15 | 4 | 22 | 9 | 25 | 21 | 16 | 4 | 2 | 1 | 0.58 | 0.31 |
| C02020301 | 18 | 12 | 27 | 21 | 12 | 22 | 8 | 6 | 3 | 2 | 5 | 17 | 19 | 12 | 6 | 23 | 20 | 1 | 16 | 9 | . | 10 | 25 | 26 | 24 | 11 | 12 | 4 | 0.58 | 0.34 |
| C01020201 | 7 | 12 | 22 | 16 | 15 | 1 | 5 | 17 | 3 | 9 | 8 | . | 11 | . | 14 | 20 | 19 | . | 13 | 5 | 10 | . | 21 | 4 | 2 | 18 | . | . | 0.59 | 0.27 |
| C14040101 | . | 2 | . | 4 | 10 | . | 13 | 1 | 3 | 7 | 14 | 15 | . | 19 | 9 | 16 | . | . | 6 | 11 | 17 | 18 | . | . | . | 5 | 12 | 8 | 0.60 | 0.31 |
| C13010202 | . | . | . | . | . | . | . | . | 4 | 5 | 6 | . | . | 7 | 8 | . | . | . | . | . | 3 | 2 | . | . | . | . | . | 1 | 0.60 | 0.37 |
| C15010221 | 26 | 21 | 1 | 15 | 23 | 4 | 7 | 3 | 25 | 14 | 18 | 9 | 13 | 20 | 16 | 2 | 5 | 12 | 6 | 17 | 8 | 22 | 19 | 11 | 24 | 28 | 27 | 10 | 0.60 | 0.26 |
| C03040201 | 19 | 25 | 8 | 23 | 1 | 16 | 10 | 21 | 22 | 18 | 17 | 2 | 24 | 4 | 7 | 27 | 28 | 11 | 3 | 14 | 11 | 13 | 8 | 15 | 26 | 5 | 20 | 6 | 0.60 | 0.22 |
| C15060101 | 9 | 9 | 9 | 9 | 23 | 9 | 1 | 9 | 1 | 9 | 1 | 23 | 9 | 20 | 23 | 23 | 23 | 20 | 1 | 1 | 9 | 9 | . | 9 | 20 | 1 | 1 | 1 | 0.60 | 0.36 |
| C01020101 | 11 | 6 | . | . | . | 1 | 1 | 21 | 3 | 8 | 13 | 17 | 23 | 18 | 10 | 22 | 20 | 15 | . | 7 | 12 | 13 | . | 5 | 4 | 19 | 9 | 16 | 0.61 | 0.30 |
| C04050151 | 6 | 15 | 25 | 16 | 3 | 12 | 4 | 2 | 1 | 13 | 9 | 22 | . | 9 | 5 | 11 | 19 | 21 | . | 7 | 8 | 20 | 23 | 24 | 17 | 18 | 14 | . | 0.61 | 0.24 |
| C15040101 | 19 | 21 | 1 | 17 | 6 | 2 | 22 | 22 | 3 | 8 | 10 | 9 | 11 | 16 | 18 | 22 | 22 | 22 | 22 | 22 | 4 | 15 | 12 | 5 | 13 | 20 | 14 | 7 | 0.61 | 0.39 |
| C17010103 | 5 | 4 | 23 | 8 | 17 | 18 | 1 | 20 | 3 | 2 | 10 | 7 | 13 | 9 | 28 | 24 | 26 | 11 | 25 | 14 | 16 | 15 | 27 | 19 | 12 | 21 | 6 | 22 | 0.61 | 0.23 |
| C06020101 | 1 | 15 | 25 | 27 | 24 | . | 11 | 10 | 18 | 23 | 3 | 11 | 16 | 6 | 19 | 19 | 9 | 3 | 11 | 3 | 14 | 17 | 19 | 22 | 25 | 6 | 8 | 2 | 0.62 | 0.28 |
| C04010121 | 16 | 12 | 26 | 13 | 25 | 10 | . | 1 | 3 | 11 | 9 | 22 | 19 | 15 | 2 | 14 | 18 | 20 | 23 | 17 | 4 | 8 | 24 | 21 | 6 | . | 7 | 5 | 0.63 | 0.26 |
| C03020101 | 10 | 18 | 26 | 23 | . | 7 | 12 | 2 | 2 | 20 | 12 | 18 | 15 | 6 | 9 | 15 | 10 | 5 | 25 | 15 | 22 | 12 | 27 | 24 | 1 | 8 | 4 | 20 | 0.63 | 0.26 |
| C08060101 | 8 | 16 | 26 | 22 | 25 | 4 | 9 | 6 | 13 | 20 | 4 | 24 | 21 | 28 | 17 | 10 | 15 | 3 | 14 | 1 | 12 | 11 | 27 | 18 | 7 | 23 | 2 | 19 | 0.63 | 0.23 |
| C08080111 | 22 | 8 | 25 | 11 | 10 | 16 | 4 | 21 | 7 | 16 | 5 | 6 | 12 | 14 | 15 | 26 | 27 | 23 | 19 | 1 | 9 | 24 | 28 | 18 | 20 | 13 | 2 | 3 | 0.63 | 0.22 |
| C16030111 | . | . | . | . | . | . | . | 3 | 7 | 5 | . | . | . | 2 | 1 | . | . | . | . | . | 6 | . | . | . | . | . | 4 | . | 0.63 | 0.32 |
| C02040132 | 7 | 3 | . | . | . | 13 | 4 | 22 | 16 | 1 | 2 | 15 | 11 | 12 | 6 | 20 | 19 | 10 | . | 5 | 21 | 23 | . | 17 | 14 | 18 | 8 | 9 | 0.64 | 0.29 |
| C10050111 | . | 6 | . | 2 | 14 | . | 18 | . | . | . | 5 | 9 | 16 | 11 | 7 | 3 | 15 | 8 | 13 | . | 12 | . | . | 4 | 1 | . | 10 | 17 | 0.64 | 0.19 |
| C03020201 | 27 | 15 | 25 | 23 | 21 | 5 | 17 | 1 | 3 | 17 | 13 | 16 | 12 | 7 | 11 | 13 | 9 | 6 | 28 | 19 | 19 | 9 | 26 | 23 | 2 | 7 | 3 | 21 | 0.64 | 0.22 |
| C050b0103 | . | . | . | . | . | 3 | . | . | 1 | 6 | . | . | . | . | 5 | . | 4 | . | . | . | . | . | . | . | 2 | . | . | . | 0.64 | 0.42 |
| C04060121 | 7 | . | . | . | 10 | 2 | 5 | 6 | 1 | 14 | 9 | . | . | 16 | 13 | . | . | . | . | 3 | 11 | . | . | 4 | . | 15 | 8 | 12 | 0.65 | 0.36 |
| C10040101 | 6 | 2 | 18 | 15 | 24 | 20 | 9 | 8 | 12 | 5 | 7 | 21 | 25 | 3 | 28 | 17 | 22 | 13 | 23 | 1 | 26 | 16 | 27 | 19 | 11 | 4 | 14 | 10 | 0.65 | 0.23 |
| C20020312 | 15 | 20 | 22 | 3 | 26 | 19 | 25 | 28 | 27 | 5 | 17 | 14 | 9 | 1 | 12 | 9 | 23 | 24 | 13 | 7 | 21 | 4 | 8 | 11 | 16 | 6 | 18 | 2 | 0.65 | 0.22 |
| C20020322 | 15 | 20 | 22 | 3 | 26 | 19 | 25 | 28 | 27 | 5 | 17 | 14 | 9 | 1 | 12 | 9 | 23 | 24 | 13 | 7 | 21 | 4 | 8 | 11 | 16 | 6 | 18 | 2 | 0.65 | 0.22 |
| C04060111 | 8 | . | . | . | 7 | 3 | 9 | 5 | 1 | 14 | 12 | . | . | 16 | 11 | . | . | . | . | 4 | 13 | . | . | 2 | . | 15 | 6 | 10 | 0.65 | 0.31 |
| C08080121 | 20 | 19 | 2 | 14 | 8 | 13 | 23 | 16 | 22 | 28 | 24 | 3 | 9 | 18 | 12 | 4 | 5 | 25 | 17 | 15 | 7 | 27 | 1 | 6 | 21 | 26 | 11 | 10 | 0.65 | 0.27 |
| C04010111 | 12 | 10 | 22 | 21 | . | 11 | . | 1 | 3 | 14 | 13 | 20 | . | . | 4 | 7 | 18 | 19 | . | 5 | 2 | 15 | 23 | 17 | 6 | 16 | 8 | 9 | 0.65 | 0.24 |
| C20020312 | 21 | 18 | 23 | 3 | 26 | 20 | 15 | 28 | 27 | 5 | 17 | 13 | 12 | 1 | 10 | 8 | 24 | 25 | 11 | 14 | 22 | 4 | 7 | 9 | 16 | 6 | 19 | 2 | 0.65 | 0.22 |
| **ID** | **Aut** | **Bel** | **Bgr** | **Hrv** | **Cyp** | **Cze** | **Dnk** | **Est** | **Fin** | **Fra** | **Deu** | **Grc** | **Hun** | **Ita** | **Ire** | **Lva** | **Lth** | **Lux** | **Mlt** | **Nld** | **Pol** | **Prt** | **Rou** | **Svk** | **Svn** | **Esp** | **Swe** | **Gbr** | **Average** | **St.Dev** |
| C05010131 | 1 | . | 1 | 1 | 18 | 18 | 18 | 1 | 1 | 18 | 1 | 1 | 18 | . | 18 | 1 | 1 | 1 | 18 | 1 | 1 | 1 | 1 | 1 | 18 | 1 | 1 | 18 | 0.65 | 0.49 |
| C20020312 | 14 | 27 | 4 | 6 | 2 | 8 | 22 | 11 | 25 | 21 | 17 | 3 | 13 | 24 | 9 | 23 | 26 | 1 | . | 5 | 18 | 12 | 20 | 16 | 15 | 7 | 19 | 9 | 0.66 | 0.27 |
| C14050101 | . | . | 1 | 14 | 23 | 3 | 7 | 3 | 21 | 16 | 17 | 9 | . | 18 | 15 | 6 | 13 | . | 2 | 5 | 10 | 19 | 8 | . | 20 | 11 | 22 | 12 | 0.66 | 0.28 |
| C04050161 | 5 | . | 18 | . | 10 | 13 | 2 | 8 | 6 | . | . | 14 | . | . | 4 | 12 | 15 | . | . | . | 9 | 17 | 19 | 16 | 11 | 7 | 3 | 1 | 0.66 | 0.30 |
| C09050201 | 5 | 1 | 22 | 25 | 27 | 14 | 2 | 15 | 4 | 11 | 8 | 16 | 17 | 23 | 7 | 26 | 18 | 9 | 24 | 6 | 19 | 13 | 28 | 21 | 10 | 20 | 3 | 12 | 0.66 | 1.24 |
| C01050302 | 8 | . | . | . | . | . | . | 2 | 5 | 6 | 7 | . | . | 9 | 10 | . | . | . | . | . | 4 | 3 | . | . | . | . | . | 1 | 0.66 | 0.30 |
| C04050171 | 5 | . | 20 | . | 9 | 13 | 2 | 7 | 4 | . | . | 14 | 19 | 11 | 6 | 12 | 15 | . | . | . | 8 | 17 | 18 | 16 | 10 | . | 2 | 1 | 0.66 | 0.31 |
| 060b0133 | 1 | . | . | . | . | . | . | 3 | . | . | . | . | 3 | . | . | . | 1 | . | . | 5 | . | . | . | . | . | . | . | . | 0.67 | 0.41 |
| C09040102 | 9 | 18 | 26 | 14 | 22 | 24 | 4 | 15 | 20 | 5 | 19 | 28 | 17 | 8 | 3 | 9 | 6 | 12 | 2 | 26 | 25 | 16 | 11 | 23 | 21 | 13 | 1 | 7 | 0.67 | 0.23 |
| 100c0103 | . | . | . | 5 | . | . | . | 1 | . | . | . | . | 7 | . | . | 1 | 6 | . | . | . | 4 | . | 3 | . | . | . | . | . | 0.68 | 0.37 |
| C03030401 | 13 | 5 | 13 | 6 | 22 | 16 | 26 | 16 | 28 | 1 | 8 | 16 | 16 | 22 | 1 | 21 | 8 | 8 | 16 | 1 | 1 | 6 | 25 | 22 | 27 | 8 | 13 | 8 | 0.68 | 0.27 |
| C04010221 | . | 12 | . | 1 | 7 | 3 | 17 | 16 | 11 | . | 19 | 6 | 13 | 15 | 4 | 14 | 1 | 22 | 21 | 20 | 9 | 24 | 18 | 10 | 5 | 23 | 8 | . | 0.68 | 0.31 |
| C04070131 | 19 | 14 | 17 | . | 3 | 20 | 16 | 3 | 11 | 1 | 3 | . | 8 | 14 | 11 | 3 | 9 | . | 18 | . | 9 | . | 1 | . | 11 | 3 | . | . | 0.68 | 0.26 |
| C10020122 | 7 | 4 | 28 | 15 | 19 | 2 | 5 | 14 | 6 | 17 | 11 | 16 | 18 | 23 | 13 | 25 | 26 | 20 | 12 | 10 | 9 | 21 | 24 | 1 | 2 | 22 | 7 | 27 | 0.68 | 0.21 |
| C04070121 | . | 7 | 19 | . | 4 | 22 | 18 | 13 | 14 | 1 | 6 | . | 10 | 7 | 14 | 9 | 11 | . | 12 | . | 17 | . | 1 | 20 | 3 | 5 | 16 | 21 | 0.68 | 0.26 |
| C04010211 | . | 23 | . | 12 | 7 | 6 | 9 | 15 | 1 | . | 22 | 8 | 13 | 10 | 17 | 11 | 14 | 24 | 24 | 19 | 16 | 20 | 18 | 4 | 1 | 21 | 5 | 1 | 0.68 | 0.33 |
| C03040101 | 10 | 12 | 28 | 22 | 1 | 19 | 14 | 20 | 4 | 12 | 17 | 18 | 27 | 3 | 6 | 26 | 24 | 6 | 11 | 8 | 23 | 15 | 25 | 21 | 16 | 4 | 2 | 8 | 0.69 | 0.29 |
| C140c0103 | . | . | . | 2 | . | 5 | . | 8 | . | . | . | . | . | . | 1 | . | 2 | . | . | . | . | 7 | 5 | . | 2 | . | . | . | 0.69 | 0.30 |
| C11050102 | . | . | 2 | 8 | . | 4 | . | . | . | 10 | . | . | . | 7 | 3 | . | . | . | . | . | . | . | 1 | . | 9 | 5 | . | 6 | 0.69 | 0.37 |
| C14010111 | . | 17 | 14 | 9 | . | . | 12 | 20 | 18 | 21 | 11 | 1 | . | 3 | 13 | 16 | 7 | . | 1 | 8 | 5 | 6 | 15 | . | 22 | 4 | 19 | 10 | 0.70 | 0.29 |
| C12040101 | 3 | 8 | 3 | 12 | 9 | 5 | 11 | 5 | 17 | 19 | 14 | 27 | 19 | 15 | 16 | 21 | 12 | 26 | 28 | 25 | 5 | 22 | 24 | 22 | 10 | 18 | 1 | 1 | 0.70 | 0.27 |
| C17150113 | 8 | 9 | . | . | . | . | 6 | . | 1 | 5 | 3 | . | . | 2 | 7 | . | . | . | . | . | . | 4 | 10 | . | . | . | . | . | 0.70 | 0.32 |
| C13010302 | . | . | 8 | 8 | . | 1 | . | 1 | 1 | . | . | . | . | . | 1 | . | . | . | . | 1 | 1 | . | 8 | . | 1 | . | . | . | 0.70 | 0.48 |
| C030b0133 | . | 8 | 21 | . | 10 | . | 11 | 14 | 12 | 19 | 18 | . | 6 | 16 | 7 | 17 | 9 | 20 | 2 | 15 | . | 2 | . | . | 13 | 5 | 4 | 1 | 0.70 | 0.27 |
| C15010211 | 21 | 15 | 3 | 2 | 27 | 8 | 1 | 7 | 20 | 19 | 18 | 11 | 13 | 16 | 4 | 5 | 6 | 26 | . | 12 | 9 | 22 | 23 | 14 | 17 | 25 | 24 | 10 | 0.70 | 0.29 |
| C15020141 | 17 | . | 9 | 1 | 15 | 1 | 18 | 13 | 1 | 19 | 14 | . | 7 | . | 12 | 1 | 1 | . | 20 | 11 | . | . | 21 | 1 | 8 | . | 10 | 16 | 0.71 | 0.33 |
| C16010321 | . | . | . | . | . | 9 | 6 | . | 7 | 4 | . | . | . | 3 | . | . | . | 2 | . | 1 | 7 | . | . | . | . | . | 4 | . | 0.71 | 0.31 |
| C15030101 | . | . | . | . | 16 | 9 | 8 | . | 1 | 10 | . | 14 | 11 | 11 | . | 11 | 3 | 4 | . | . | 6 | 17 | 2 | 4 | 6 | 15 | . | . | 0.71 | 0.26 |
| C030d0213 | 4 | . | . | 10 | 16 | 9 | . | . | 3 | 5 | 7 | 17 | . | 18 | 8 | 14 | 12 | 11 | 15 | 1 | 13 | . | . | . | . | . | 2 | 6 | 0.71 | 0.25 |
| C10020132 | 11 | 8 | . | . | . | 14 | 1 | 22 | 6 | 3 | 12 | 7 | 21 | 10 | 16 | 22 | 20 | 5 | . | 2 | 18 | 9 | . | 4 | 17 | 12 | 15 | 19 | 0.72 | 0.28 |
| C03090101 | 11 | 13 | 27 | 23 | 15 | 20 | 8 | 18 | 1 | 3 | 13 | 19 | 25 | 11 | 6 | 26 | 21 | 6 | 15 | 9 | 24 | 3 | . | 21 | 17 | 3 | 1 | 9 | 0.72 | 0.24 |
| C13010102 | . | . | 4 | 10 | 1 | 6 | . | . | . | 12 | 9 | . | . | . | 5 | . | . | 1 | . | . | . | . | 3 | . | 11 | 7 | . | 8 | 0.72 | 0.35 |
| **ID** | **Aut** | **Bel** | **Bgr** | **Hrv** | **Cyp** | **Cze** | **Dnk** | **Est** | **Fin** | **Fra** | **Deu** | **Grc** | **Hun** | **Ita** | **Ire** | **Lva** | **Lth** | **Lux** | **Mlt** | **Nld** | **Pol** | **Prt** | **Rou** | **Svk** | **Svn** | **Esp** | **Swe** | **Gbr** | **Average** | **St.Dev** |
| C030b0123 | 28 | 7 | 21 | 18 | 1 | 10 | 10 | 25 | 25 | 13 | 21 | 1 | 1 | 13 | 18 | 1 | 24 | 1 | 7 | 18 | 16 | 1 | 27 | 10 | 16 | 13 | 7 | 21 | 0.73 | 0.25 |
| C01050402 | . | . | 9 | 9 | . | 1 | . | 1 | 1 | . | . | . | . | . | 1 | . | . | . | . | 1 | 1 | . | 9 | 1 | 1 | . | . | . | 0.73 | 0.47 |
| C06030201 | 9 | . | 13 | 18 | 14 | 6 | 19 | 11 | 1 | 10 | . | 21 | 16 | . | 15 | 12 | . | . | 1 | 5 | 3 | . | 8 | 17 | 7 | . | 20 | 4 | 0.74 | 0.24 |
| C08050201 | 10 | 15 | 8 | 21 | 24 | 1 | 13 | 10 | 22 | 25 | 2 | 28 | 4 | 26 | 13 | 18 | 18 | 16 | 9 | 4 | 3 | 20 | 7 | 17 | 10 | 27 | 23 | 6 | 0.74 | 0.21 |
| C040a0113 | . | 1 | . | . | . | . | 1 | 1 | 1 | 1 | 1 | 16 | 1 | 1 | . | . | . | 1 | 1 | 1 | . | 1 | 1 | 1 | . | . | . | . | 0.74 | 0.45 |
| C02010201 | 12 | 26 | 1 | 18 | . | 9 | 14 | 9 | 24 | 5 | 5 | 21 | 19 | 16 | 1 | 5 | 21 | 9 | 12 | 19 | 4 | 25 | 27 | 14 | 1 | 23 | 16 | 5 | 0.75 | 0.25 |
| C04020201 | 2 | 14 | 27 | 18 | 11 | 23 | 12 | . | 15 | 1 | 8 | 20 | 24 | 10 | 6 | 13 | 16 | 4 | 9 | 4 | 17 | 22 | 26 | 25 | 21 | 19 | 3 | 6 | 0.75 | 0.29 |
| C110b0102 | . | . | 4 | . | . | . | . | 3 | 7 | 8 | 9 | . | . | . | 10 | 1 | . | . | . | . | 6 | 4 | . | . | . | . | . | 1 | 0.76 | 0.30 |
| C03090201 | 9 | 23 | 9 | 9 | 23 | 16 | 23 | 1 | 1 | 23 | 27 | 1 | 16 | 9 | 9 | 1 | 9 | 1 | 1 | 16 | 9 | 16 | . | 1 | 1 | 16 | 16 | 16 | 0.76 | 0.23 |
| C05010121 | 21 | . | 7 | 1 | 11 | 11 | 8 | 26 | 25 | 11 | 11 | 21 | 21 | . | 18 | 18 | 1 | 18 | 1 | 21 | 11 | 8 | 11 | 1 | 1 | 1 | 10 | 11 | 0.76 | 0.26 |
| C03010101 | 9 | 9 | 25 | 20 | 15 | 1 | 6 | 24 | 1 | 20 | 17 | 1 | 26 | 1 | 9 | 27 | 20 | 9 | 15 | 9 | 1 | 20 | 27 | 9 | 17 | 6 | 6 | 17 | 0.76 | 0.26 |
| C03010201 | 13 | . | . | 1 | 17 | . | 19 | 11 | 1 | 16 | 15 | 1 | 9 | 1 | 9 | 1 | 1 | . | 7 | . | 7 | 12 | 18 | 14 | . | . | . | . | 0.76 | 0.33 |
| C06040201 | 12 | 27 | 25 | 2 | 20 | 19 | 21 | 16 | 9 | 18 | 24 | 17 | 11 | 22 | 10 | 1 | 3 | 6 | 28 | 15 | 23 | 13 | 7 | 4 | 8 | 26 | 5 | 14 | 0.77 | 0.23 |
| C01050102 | 11 | . | 5 | 12 | 1 | 7 | . | . | . | 14 | . | . | . | 10 | 6 | . | . | 1 | . | 1 | . | . | 4 | . | 13 | 8 | . | 9 | 0.77 | 0.33 |
| C20020322 | 11 | 12 | 25 | 16 | 22 | 20 | 9 | . | 15 | 5 | 10 | 18 | 21 | 4 | 1 | 19 | . | 2 | . | 7 | 24 | 13 | 23 | 17 | 14 | 8 | 6 | 3 | 0.77 | 0.23 |
| C120b0103 | 11 | 12 | 25 | 16 | 22 | 20 | 9 | . | 15 | 5 | 10 | 18 | 21 | 4 | 1 | 19 | . | 2 | . | 7 | 24 | 13 | 23 | 17 | 14 | 8 | 6 | 3 | 0.77 | 0.23 |
| C03070201 | 9 | 9 | 28 | 15 | 12 | 20 | 1 | 18 | 6 | 15 | 13 | 15 | 25 | 4 | 11 | 23 | 21 | 7 | 23 | 2 | 19 | 14 | 27 | 26 | 3 | 8 | 5 | 22 | 0.77 | 0.25 |
| C110b0202 | . | . | 8 | . | . | 1 | . | 1 | 1 | . | . | . | . | . | 1 | . | . | . | . | 1 | 1 | . | 8 | . | 1 | . | . | . | 0.78 | 0.44 |
| C01050202 | . | . | 7 | 10 | 5 | 8 | . | 2 | 3 | 4 | . | . | . | . | 1 | . | . | . | . | . | . | . | 6 | . | 9 | . | . | . | 0.78 | 0.31 |
| C08100121 | 8 | 7 | 27 | 21 | 19 | 25 | 1 | 9 | 2 | 14 | 5 | 22 | 26 | 15 | 13 | 17 | 24 | 6 | 11 | 4 | 20 | 18 | 28 | 23 | 10 | 16 | 3 | 12 | 0.79 | 0.24 |
| C09030201 | . | . | . | . | 4 | . | 6 | . | . | . | . | 1 | 9 | 1 | 7 | . | . | 10 | . | 1 | . | . | . | 8 | 11 | . | 5 | . | 0.79 | 0.24 |
| C16010101 | 11 | . | 18 | 3 | 16 | . | 12 | 22 | 19 | 15 | 10 | 9 | 8 | 4 | 21 | 24 | 23 | 2 | 20 | 4 | 7 | . | 17 | 14 | 1 | 6 | 13 | . | 0.79 | 0.23 |
| C03030101 | . | . | 10 | 1 | . | 10 | 1 | 19 | 8 | 16 | 5 | 10 | 1 | 10 | 15 | 18 | . | 16 | . | 5 | . | 5 | 8 | 1 | . | 14 | . | . | 0.80 | 0.27 |
| C060b0113 | 1 | . | . | . | . | . | . | 1 | . | . | . | . | 1 | . | . | . | 1 | . | . | 5 | . | . | . | . | . | . | . | . | 0.80 | 0.45 |
| C05060221 | . | 1 | . | . | . | 14 | 9 | . | 1 | . | 9 | 12 | . | . | 1 | 12 | 9 | . | 1 | 1 | . | . | . | . | 1 | . | 1 | 1 | 0.80 | 0.33 |
| C14010121 | . | 11 | 14 | 6 | . | . | 9 | . | . | 13 | 4 | 3 | . | 8 | 1 | . | . | 10 | . | 2 | . | 7 | . | . | . | 12 | . | 5 | 0.81 | 0.29 |
| C04070111 | 18 | 12 | 19 | . | 1 | 17 | . | 13 | 13 | 1 | 1 | . | 1 | 13 | 13 | 1 | 1 | . | . | . | 1 | . | 1 | 20 | 1 | 1 | 1 | 21 | 0.81 | 0.30 |
| C02020101 | 3 | 3 | 27 | 3 | 3 | 26 | 3 | 3 | 3 | 3 | 1 | 2 | 3 | 3 | 3 | 3 | 3 | 3 | 3 | 3 | 3 | 3 | . | 3 | 3 | 3 | 3 | 3 | 0.81 | 0.16 |
| C14010121 | 1 | 8 | 27 | 22 | 9 | . | 1 | 9 | 9 | 9 | 9 | 9 | 17 | 1 | 25 | 24 | 23 | 17 | 1 | 17 | 1 | 1 | 26 | 17 | 17 | 1 | 9 | 9 | 0.81 | 0.28 |
| C04070141 | . | 1 | 18 | . | 19 | . | 11 | 11 | . | 1 | 9 | . | 17 | 11 | 11 | 1 | 1 | . | 10 | . | 1 | . | 1 | 20 | 1 | 1 | 11 | 11 | 0.82 | 0.24 |
| C11050202 | . | . | 8 | 6 | 5 | 9 | . | 2 | 3 | 4 | . | . | . | . | 1 | . | . | . | . | . | . | . | 7 | . | 10 | . | . | . | 0.82 | 0.30 |
| C06030101 | 4 | 14 | 22 | 26 | 24 | 16 | 6 | 15 | 13 | 12 | 2 | 11 | 17 | 8 | 19 | 10 | 9 | 5 | 27 | 1 | 20 | 23 | . | 21 | 25 | 18 | 7 | 3 | 0.82 | 0.21 |
| **ID** | **Aut** | **Bel** | **Bgr** | **Hrv** | **Cyp** | **Cze** | **Dnk** | **Est** | **Fin** | **Fra** | **Deu** | **Grc** | **Hun** | **Ita** | **Ire** | **Lva** | **Lth** | **Lux** | **Mlt** | **Nld** | **Pol** | **Prt** | **Rou** | **Svk** | **Svn** | **Esp** | **Swe** | **Gbr** | **Average** | **St.Dev** |
| C030d0103 | 4 | . | . | 15 | 18 | 10 | . | . | 3 | 10 | 6 | 17 | . | 16 | 13 | 9 | 7 | 5 | 14 | 1 | 12 | . | . | . | . | . | 2 | 8 | 0.82 | 0.24 |
| C16020201 | 12 | 13 | 20 | 5 | 5 | 8 | 15 | 2 | 16 | 27 | 23 | 11 | 22 | 25 | 14 | 3 | 9 | . | 7 | 26 | 19 | 17 | 21 | 9 | 4 | 18 | 1 | 24 | 0.83 | 0.23 |
| C03090301 | 5 | 16 | 21 | 16 | 1 | 16 | 1 | 25 | 16 | 10 | 10 | 5 | 21 | 10 | 10 | 27 | 26 | 5 | 1 | 1 | 21 | 10 | 28 | 21 | 5 | 16 | 5 | 10 | 0.83 | 0.19 |
| C01010101 | 14 | 5 | 26 | 20 | 1 | 1 | 5 | 14 | 5 | 1 | 8 | 23 | 21 | 26 | 8 | 22 | 25 | 14 | 8 | 8 | 14 | 19 | 28 | 8 | 1 | 23 | 8 | 14 | 0.83 | 0.21 |
| C15030101 | 15 | 4 | 8 | 3 | 14 | 9 | . | . | . | . | . | . | 1 | 11 | 13 | . | . | 2 | . | . | 12 | . | 5 | 7 | 6 | 10 | 17 | 16 | 0.84 | 0.25 |
| C10070301 | . | . | . | 6 | . | . | . | . | . | 9 | . | 11 | . | 10 | 1 | . | . | . | 8 | . | . | 1 | 7 | . | 1 | 12 | 1 | 1 | 0.84 | 0.30 |
| C130a0103 | 13 | 15 | 10 | 7 | 2 | 16 | 9 | 4 | . | 18 | 19 | . | 12 | . | 11 | 3 | 6 | . | 1 | . | 17 | . | 14 | 8 | 5 | . | . | . | 0.84 | 0.25 |
| C06050201 | 1 | 1 | 18 | 1 | . | 1 | 1 | 1 | 1 | 21 | 1 | 24 | . | 1 | 1 | 20 | 23 | 1 | . | 1 | 22 | 1 | 1 | 19 | 1 | 1 | 1 | 25 | 0.85 | 0.28 |
| C10050121 | . | 15 | 19 | 6 | 23 | 9 | 17 | 18 | . | 14 | 7 | 24 | 1 | 22 | 13 | 21 | 5 | 4 | 16 | 20 | 8 | 11 | . | 10 | 3 | 11 | 2 | . | 0.85 | 0.22 |
| C11010101 | . | . | . | . | . | . | 10 | . | . | . | 8 | 7 | 1 | . | . | 4 | . | . | . | . | 8 | 6 | . | . | 5 | 2 | 10 | 3 | 0.86 | 0.33 |
| C05060241 | . | 1 | . | . | . | 1 | 12 | . | 1 | . | 1 | 12 | . | . | 1 | 1 | 14 | . | 1 | 1 | . | . | . | . | 1 | . | 1 | 1 | 0.86 | 0.31 |
| C030b0113 | 24 | 9 | 15 | . | 21 | . | 3 | . | 14 | 11 | 19 | 5 | 1 | 11 | 17 | 18 | 20 | 5 | . | 10 | 23 | 2 | 15 | 5 | 22 | 8 | 3 | 13 | 0.86 | 0.21 |
| C03030201 | 10 | 17 | 24 | 16 | 12 | 4 | 6 | 21 | 3 | 18 | 14 | 1 | 12 | 10 | 9 | 25 | 27 | 18 | 25 | 5 | 22 | 23 | 28 | 2 | 7 | 20 | 8 | 15 | 0.86 | 0.22 |
| C06010101 | 11 | 1 | 15 | . | 1 | . | 18 | 20 | 1 | 11 | 1 | 1 | 25 | 20 | 18 | 20 | 23 | 11 | 1 | 1 | 15 | 23 | 26 | 11 | 15 | 1 | 1 | 1 | 0.87 | 0.21 |
| C11060301 | 1 | 1 | . | . | . | 10 | 1 | . | 1 | 1 | 1 | . | 16 | 11 | 14 | . | . | . | . | 1 | 15 | 13 | . | . | . | 12 | 1 | 9 | 0.87 | 0.26 |
| C10050131 | . | 11 | 21 | 23 | 24 | 8 | 6 | 10 | . | 12 | 4 | 25 | 7 | 22 | 16 | 19 | 3 | 2 | 15 | 9 | 18 | 20 | . | 13 | 16 | 14 | 1 | 5 | 0.87 | 0.21 |
| C12070101 | 1 | 1 | . | 1 | . | 15 | 1 | . | 1 | 1 | 1 | . | 15 | 1 | 1 | . | . | . | . | 1 | 1 | . | . | 1 | . | 1 | 1 | . | 0.88 | 0.34 |
| C090c0103 | 23 | 1 | 7 | 15 | 14 | 9 | 1 | . | 8 | 18 | 9 | 18 | 17 | 1 | 27 | 26 | 18 | 23 | 1 | 18 | 1 | 9 | 22 | 25 | 12 | 16 | 1 | 12 | 0.88 | 0.21 |
| C03030501 | 23 | 21 | 28 | 10 | 1 | 1 | 1 | 1 | 18 | 26 | 27 | 1 | 1 | 10 | 1 | 15 | 25 | 10 | 10 | 1 | 24 | 19 | 1 | 19 | 10 | 17 | 22 | 15 | 0.88 | 0.23 |
| C05060231 | . | 1 | . | . | . | 1 | 1 | . | 1 | . | . | 11 | . | . | . | 1 | 1 | . | 1 | 1 | . | . | . | . | . | . | 1 | 1 | 0.91 | 0.30 |
| C14010111 | 1 | 1 | 23 | . | 1 | . | 1 | 1 | 1 | 1 | 1 | 1 | 1 | 1 | 26 | 23 | 25 | 1 | 1 | 1 | 1 | 1 | 1 | 1 | 1 | 1 | 1 | 1 | 0.91 | 0.24 |
| C15080101 | 1 | 1 | 25 | 1 | 28 | 1 | 1 | 1 | 1 | 1 | 1 | 1 | 1 | 25 | 1 | 25 | 1 | 1 | 1 | 1 | 1 | 1 | 1 | 1 | 1 | 1 | 1 | 1 | 0.91 | 0.24 |
| C01030101 | 1 | 1 | 24 | . | 1 | 22 | 1 | 1 | 1 | 1 | 1 | . | 1 | . | 1 | 1 | 23 | 1 | . | 1 | 1 | 1 | 1 | 1 | 1 | 1 | 1 | 1 | 0.92 | 0.28 |
| C040a0143 | . | 1 | . | . | . | . | 1 | 1 | 1 | 1 | . | . | . | . | 1 | 1 | . | . | . | 1 | 1 | 1 | . | 13 | 1 | 1 | . | . | 0.92 | 0.28 |
| C040a0133 | . | 1 | . | . | . | . | 1 | 1 | 1 | 1 | 1 | . | 1 | 20 | 1 | 1 | . | . | . | 1 | 1 | 1 | . | 1 | 1 | 1 | . | . | 0.94 | 0.25 |
| C02010101 | 1 | 1 | 27 | 1 | 1 | 1 | 1 | 1 | 1 | 1 | 1 | 1 | 1 | 1 | 1 | 1 | 1 | 1 | 1 | 1 | 1 | 1 | 1 | 28 | 1 | 1 | 1 | 1 | 0.95 | 0.20 |
| C10070401 | 12 | 9 | 21 | 27 | 17 | 23 | 8 | 24 | 1 | 2 | 4 | 14 | 26 | 7 | 6 | 22 | 19 | 13 | 16 | 11 | 18 | 10 | 20 | 25 | 15 | . | 3 | 5 | 0.95 | 0.19 |
| C07010201 | 1 | 1 | . | 1 | 1 | 1 | 1 | 1 | 1 | 1 | 1 | 1 | 1 | 1 | 1 | 1 | 1 | 1 | 1 | 1 | 1 | 1 | 27 | 1 | 1 | 1 | 1 | 1 | 0.96 | 0.19 |

Table S3 EU-27 Composite Index Scores using the arithmetic, geometric, and harmonic mean

| Country | Arithmetic | Geometric | Harmonic |
| --- | --- | --- | --- |
| AUT | 0.624 | 0.476 | 0.169 |
| BEL | 0.643 | 0.493 | 0.225 |
| BGR | 0.433 | 0.342 | 0.004 |
| CYP | 0.522 | 0.370 | 0.000 |
| CZE | 0.576 | 0.408 | 0.000 |
| DNK | 0.666 | 0.541 | 0.003 |
| DEU | 0.660 | 0.550 | 0.220 |
| ESP | 0.595 | 0.459 | 0.117 |
| EST | 0.596 | 0.460 | 0.005 |
| FIN | 0.655 | 0.457 | 0.012 |
| FRA | 0.608 | 0.540 | 0.069 |
| GBR | 0.655 | 0.561 | 0.148 |
| GRC | 0.488 | 0.302 | 0.000 |
| HRV | 0.583 | 0.427 | 0.001 |
| HUN | 0.528 | 0.365 | 0.019 |
| IRE | 0.617 | 0.441 | 0.098 |
| ITA | 0.571 | 0.444 | 0.085 |
| LTU | 0.531 | 0.382 | 0.001 |
| LVA | 0.520 | 0.398 | 0.005 |
| LUX | 0.593 | 0.389 | 0.010 |
| MLT | 0.565 | 0.416 | 0.002 |
| NLD | 0.654 | 0.430 | 0.103 |
| POL | 0.586 | 0.415 | 0.011 |
| PRT | 0.576 | 0.370 | 0.010 |
| ROU | 0.416 | 0.307 | 0.000 |
| SVK | 0.526 | 0.337 | 0.001 |
| SVN | 0.609 | 0.389 | 0.000 |
| SWE | 0.684 | 0.560 | 0.041 |
